# Identification of PCSK9-like human gene knockouts using metabolomics, proteomics, and whole-genome sequencing in a consanguineous population

**DOI:** 10.1016/j.xgen.2022.100218

**Published:** 2022-11-15

**Authors:** Aziz Belkadi, Gaurav Thareja, Fatemeh Abbaszadeh, Ramin Badii, Eric Fauman, Omar M.E. Albagha, The Qatar Genome Program Research Consortium, Karsten Suhre

**Affiliations:** 1 Bioinformatics Core, Weill Cornell Medicine-Qatar, Education City, Doha 24144, Qatar; 2 Department of Biophysics and Physiology, Weill Cornell Medicine, New York, NY, USA; 3 Hamada Medical Corporation, Doha, Qatar; 4 Pfizer, Boston, MA, USA; 5 College of Health and Life Sciences, Hamad Bin Khalifa University, Doha, Qatar; 6 Centre for Genomic and Experimental Medicine, Institute of Genetics and Cancer, University of Edinburgh, Edinburgh, UK

**Keywords:** human gene knockouts, metabolomics, proteomics, whole-genome sequencing, consanguineous population, drug target validation, drug target identification

## Abstract

Natural human knockouts of genes associated with desirable outcomes, such as *PCSK9* with low levels of LDL-cholesterol, can lead to the discovery of new drug targets and treatments. Rare loss-of-function variants are more likely to be found in the homozygous state in consanguineous populations, and deep molecular phenotyping of blood samples from homozygous carriers can help to discriminate between silent and functional variants. Here, we combined whole-genome sequencing with proteomics and metabolomics for 2,935 individuals from the Qatar Biobank (QBB) to evaluate the power of this approach for finding genes of clinical and pharmaceutical interest. As proof-of-concept, we identified a homozygous carrier of a very rare *PCSK9* variant with extremely low circulating PCSK9 levels and low LDL. Our study demonstrates that the chances of finding such variants are about 168 times higher in QBB compared with GnomAD and emphasizes the potential of consanguineous populations for drug discovery.

## Highlights

• Studied 3,000 individuals from a highly consanguineous Arab population
• Found homozygous protein-changing variants associated with extreme protein/metabolite levels
• Compared with non-consanguineous populations, 168 times more likely to identify human knockouts
• Identified Qatar exclusive rare homozygous PCSK9 variants with low LDL-C

## Graphical abstract

**Figure:**
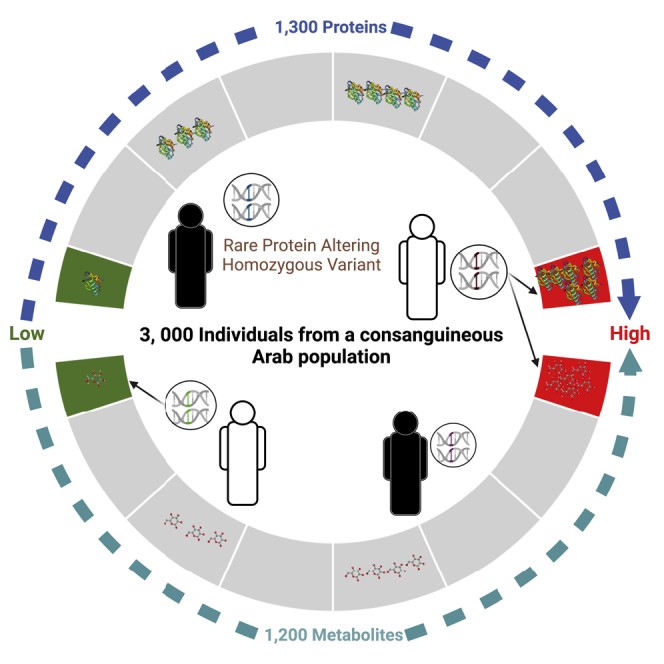

## Teaser

Belkadi et al. combined whole-genome sequencing with proteomics and metabolomics in a highly consanguineous Arab population to discover rare homozygous protein-changing variants coinciding with extreme protein and metabolite levels. We found that the chance of identifying such variants is 168 times higher than in a non-consanguineous population.

## Introduction

Cholesterol-lowering drugs that target PCSK9 are a well-documented example of how drug target selection based on genetic evidence from human knockouts can contribute to technical and regulatory success, providing a strong rationale for further investment in the field.^1^ PCSK9 inhibitors, such as alirocumab and evolocumab,^2^^,^^3^ were developed following the identification of healthy individuals with low levels of low-density lipoprotein cholesterol (LDL-C) carrying PCSK9 protein-changing variants (PCVs).^4^ The success of this approach led to the widespread implementation of attempts to identify drug targets from healthy homozygous PCV carriers with extreme protein or metabolite levels and/or extreme biochemical findings in well-phenotyped cohorts.^5^ Other successes achieved with similar strategies include LPA for lowering plasma lipoprotein levels, which was identified with biochemical assays,^6^ APOC3 for lowering plasma triglyceride concentration, which was identified from proteomics and clinical biochemistry data,^7^ and more recently HAO1 as a therapeutic target for primary hyperoxaluria type 1, identified through metabolomics.^8^

The efficacy of studies of large genotyped or sequenced population cohorts for identifying PCVs for drug discovery was recently highlighted by a study of 141,456 whole-exome sequences from the GnomAD project^9^ and 200,000 whole-exome sequences from the UK Biobank (UKB).^10^ The availability of electronic health records and the continually decreasing costs of DNA sequencing have played an important role in this success. However, despite rigorous automatic filtering and manual curation to remove common model errors,^11^ many false positives remain for rare PCVs, and it is difficult to distinguish true PCVs from processing artifacts in these sets of data from non-phenotyped subjects.^12^

Rare PCVs differ considerably between populations with different structures.^13^ In consanguineous populations with high rates of homozygosity, such as that of Qatar, the expected frequency of homozygotes for PCVs for the median gene is estimated at five per million, whereas this frequency is estimated at six per billion in non-consanguineous populations.^9^ The sequencing of 7 billion non-consanguineous subjects would not be sufficient to find a natural knockout for every human gene, whereas predictions suggest that this target could be achieved with only a few million consanguineous individuals.^9^ Consanguineous populations therefore provide a unique opportunity to identify rare homozygous PCVs, potentially leading to new PCKS9-like drug target discoveries.

Here we used deep (30x) whole-genome sequencing data from 2,935 subjects included in the Qatar Biobank (QBB)^14^^,^^15^ to identify PCVs coinciding with extreme blood circulating levels of proteins and metabolites. We identified 98 *in-cis* protein associations, for which the PCV was in the gene encoding the protein measured in blood, and 105 metabolite associations for which the affected gene was biochemically related to the blood metabolite, which could also be viewed as a biochemical *in-cis* association. We manually curated the identified associations and selected 12 cases in which the mutated genes affected the corresponding protein and metabolite levels for further in-depth investigation as potential drug targets (*PCSK9*, *BHMT*, *ACY1*, *PLG*, *ACSM2A*, *ABCG5*, *ABCC2*, *PAOX*, *AFMID*, *UPB1*, *AOX1,* and *ALOX15*). The remaining PCV-extreme phenotype associations are presented in the supplementary tables.

Interestingly, we identified two homozygous *PCSK9* variants associated with low PCSK9 protein and LDL-C levels: the first was the original rs11591147 variant that led to the development of PCSK9 inhibitors,^16^ whereas the second (rs746442570) is reported here for the first time in the homozygous state. We also discovered two *ACY1* variants affecting both protein (ACY1) and related metabolite (acetylated amino acids) levels and one *PLG* variant possibly associated with excessive coagulation of the blood, which was identified in an individual already on warfarin treatment according to the responses given on medical questionnaires. The PCV-extreme phenotype associations reported here, thus, shed new light on the functions of the proteins affected and may support further drug target development.

## Results

### The population of Qatar displays enrichment in rare PCVs

**Figure 1:**
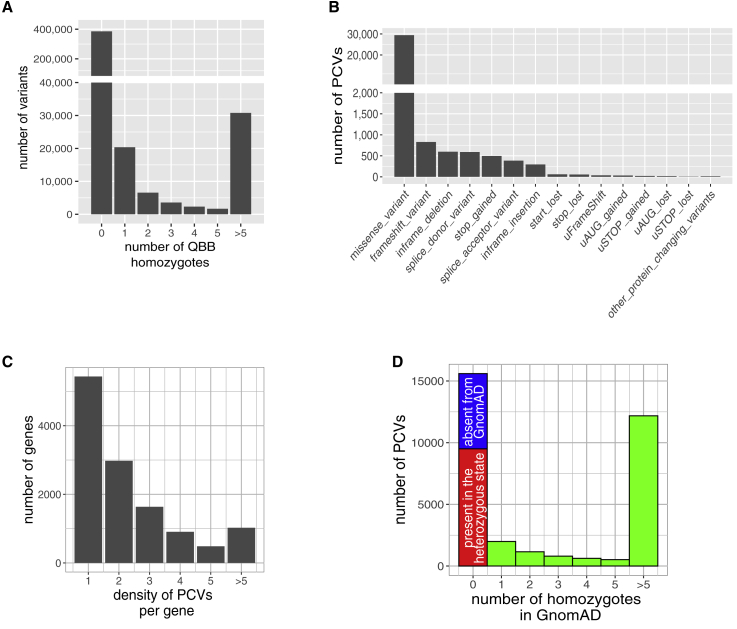
Characteristics of potential protein-changing variants (PCVs) identified in QBB (A) Distribution of homozygous variants (exonic and 5′ UTR variants with high or moderate effects) annotated by the Variant Effect Predictor (VEP) for 2,935 QBB participants. For most of the variants identified, there was no homozygote in QBB. (B) Annotation classes of 32,868 PCVs present in the homozygous state in at least one, but no more than five QBB participants. (C) Distribution of 32,868 PCVs per 12,466 genes. Most of the genes carry a single rare PCV in the homozygous state. (D) Distribution of homozygous PCVs over 125,748 participants of the GnomAD project. Almost half the PCVs identified in at least one, but not more than five QBB participants were not found in the homozygous state in GnomAD. The PCVs completely absent from GnomAD are shown in blue, those present in GnomAD exclusively in the heterozygous state are shown in red, and those identified in the homozygous state in GnomAD are shown in green.

We consider homozygous gene variants that alter the encoded protein and are rare in the population to be candidate human knockouts. After quality control filtering, we identified 32,868 exonic and 5′ UTR variants (PCVs) of 12,466 different genes that (1) had a moderate or high impact on the encoded protein, (2) were present in the homozygous state in at least one, but no more than five individuals from the 2,935 subjects included in QBB, and (3) had a minor allele frequency in the GnomAD populations <0.05 (Figure 1A). These PCVs belong to 15 classes, with missense variants the largest group, followed by frameshift variants, and then in-frame deletions (Figure 1B). Almost half the 12,466 genes (5,072, 46%) carried a single PCV (Figure 1C). The density of PCVs per gene was correlated with gene length, such that the largest genes carried the largest numbers of PCV. For example, two large human genes, *TTN* and *MUC4*, carried 111 and 493 variants, respectively.

Almost half (15,600, 47%) of the PCVs identified in QBB were not found in the homozygous state in 125,748 subjects from the different populations of the GnomAD project (Figure 1D). In total, 9,505 (61%) of these PCVs were present exclusively in the heterozygous state in GnomAD, and the rest (6,095, 39%) were completely undetected. We then split the identified PCVs into two groups: (1) 2,440 high-impact PCVs, and (2) 31,292 moderate-impact PCVs. We observed a high enrichment for the high-impact PCVs in those that were absent from the GnomAD project (Figure S1). Most of the high-impact PCVs (92%) were not detected in the GnomAD project from which only 19% were detected in the heterozygote state in the GnomAD project. Only 43% of the moderate-impact PCVs were absent from the GnomAD project.

**Figure 2:**
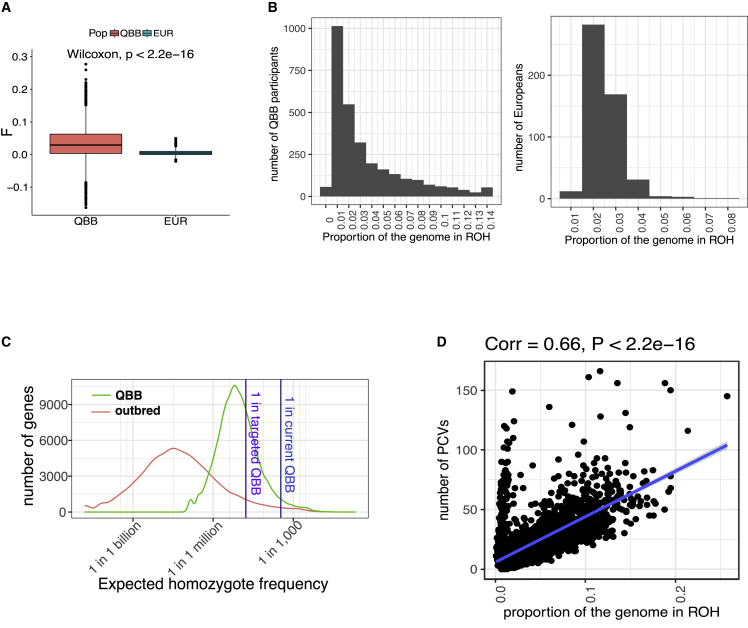
The high rate of consanguinity in QBB results in high homozygosity (A) Distribution of the inbreeding coefficient (*F*) in QBB participants and in the non-consanguineous European population of the 1000 Genomes Project. The F coefficients in Europeans were compared with that for QBB in Wilcoxon tests. (B) The proportion of the genome covered by ROH (PGROH) in 2,935 QBB participants (left) and 503 Europeans of the 1000 Genomes Project (right). (C) Distribution of the number of PCVs as a function of the proportion of the genome covered by ROH in QBB participants. Each dot represents a QBB participant. PGROH is indicated on the x axis, and the number of PCVs identified for this QBB participant is indicated on the y axis. (D) Distribution of genes by expected homozygote frequency (the smallest number of individuals required to identify at least one homozygote) in QBB (green line) and the GnomAD non-consanguineous population (red line). Sequencing a few million QBB participants would be expected to result in the identification of at least one homozygote for a loss-of-function variant for all human genes. The dark blue vertical line indicates the expected homozygote frequency for the current QBB population of 2,935 participants. The purple vertical line indicates the expected homozygote frequency for the targeted QBB population size of 60,000 participants.

We estimated the excess homozygosity in QBB by comparing the inbreeding coefficient F for QBB cohort with that for the Europeans of the 1000 Genomes Project (Figure 2A). The F coefficient describes the probability that two alleles at a locus are identical by descent and can be used to estimate the excess homozygosity in a consanguineous population relative to a non-consanguineous ancestor. The individuals included in QBB had an F value six times higher, on average, than that for non-consanguineous populations (0.033 versus 0.0052, p < 2.2e-16).

Long runs of homozygosity (ROH) generated by recent consanguinity enable rare deleterious variants to exist in the homozygous form.^17^^,^^18^ We identified long ROH exceeding 10 kb in QBB and determined the fraction of PCVs lying in these long ROH. We found that 37,093 (65%) of the 57,063 homozygous PCVs were located in long ROH. The mean proportion of genomes in long ROH (PGROH) in QBB was 3.52% (Figure 2B). For comparison, the mean PGROH in QBB was 40% higher than that for the Europeans of the 1000 Genomes Project (2.5%, Figure 2B). In total, 593 (20%) QBB participants have a PGROH above 5.8%, the PGROH observed in self-reported second cousins or closer parents.^19^ PGROH reaches this level in fewer than 1% of Europeans (Figure 2B). PGROH was also correlated with the number of homozygous PCVs carried by individuals included in QBB (Pearson’s coefficient = 0.66, p < 2.2e-16, Figure 2C).

The expected homozygote frequency (EHF) in QBB was estimated at seven per million for the median gene, a value 168 times higher than that for the non-consanguineous populations of GnomAD (Figure 2D). Based on this estimate of EHF, the targeted QBB sample size of 60,000 participants will make it possible to identify at least one homozygote for 5,709 genes, a result comparable to that obtained by sequencing five million non-consanguineous individuals (Figure 2D). Thus, the high level of homozygosity in QBB, due to recent common ancestors, provides a much higher relative power for identifying rare PCVs than can be attained with non-consanguineous populations.

### Extreme metabolomic and proteomic phenotypes identify functional variants

**Figure 3:**
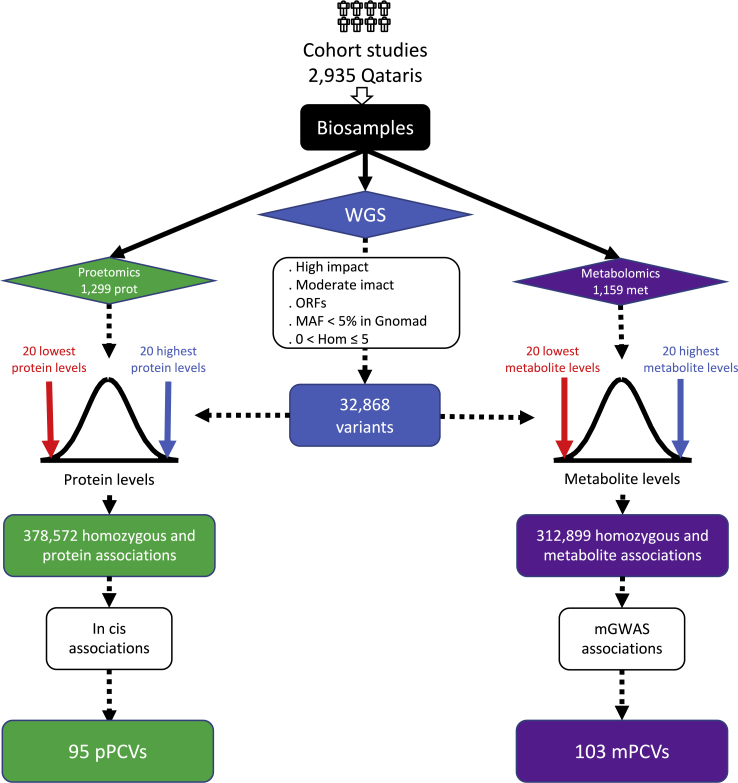
Schematic view of the study design for pPCV and mPCV analysis All samples were collected exclusively from Qatari participants of the QBB study. Variants detected from whole-genome sequencing data were annotated with the Variant Effect Predictor (VEP), and variants annotated as high-impact, moderate-impact, and as creating new upstream open reading frames (ORFs) were retained as functional variants. Common variants in the GnomAD project (MAF > 5%) were excluded. Only rare homozygous variants present in at least one but no more than five participants were included. Associations between homozygous variants and extreme (in the top 20 or the bottom 20 values) protein and metabolite levels were identified. For each variant, all homozygotes had to be ranked in the top 20 or the bottom 20 values to be retained for the analysis. We retained only *in-cis* protein associations: 95 protein-changing variants affecting protein levels (pPCVs) and 103 gene-metabolite pairs reported in genome-wide association studies on metabolites (mPCVs).

We characterized the 32,868 PCVs that were homozygous in one to five individuals in QBB further, by determining the levels of 1,305 blood-circulating proteins using the SomaLogic (Boulder, CO) affinity proteomics platform, and of 1,159 metabolites in plasma using the Metabolon (Durham, NC) non-targeted metabolomics platform. We also included data from 71 clinical biochemistry assays (Table S1) and the self-reported medication taken by these QBB participants. We assessed the association of homozygous variants with extreme protein and metabolite levels (Figure 3). We retained all variants for which all homozygous individuals had protein or metabolite levels ranking in the 20 highest or 20 lowest values for the 2,935 QBB participants. In other words, all individuals homozygous for these rare PCVs had extreme phenotypic traits. We identified 378,572 variant-extreme protein-level associations (grouped in 377,249 variant-protein pairs, Table S2) and 312,899 variant-extreme metabolite-level associations (grouped in 311,637 variant-metabolite pairs, Table S3).

We estimated the false discovery rate (FDR) of our approach by sampling, by repeating the analysis 100 times with randomized sample ids. The mean number of trait-variant pairs found by chance was 370,384 for proteins and 296,643 for metabolites, suggesting that more than 95% of such associations (for either proteins or metabolites) would be expected to be found on average by chance (Figures S2A and S2B).

As a means of reducing the FDR and focusing on the variant-trait pairs most likely to be of clinical relevance, we therefore limited our analysis to variants of genes functionally linked to the trait. For proteins, we retained only *in-cis* protein associations (pPCVs), that is, variants affecting the protein determined. We identified 95 pPCVs for 63 proteins associated with 72 variants (grouped in 73 variant-protein pairs, Table S4). The FDR for these pPCVs, determined by sampling, was 15% (Figure S2C).

For metabolites, we limited the analysis to metabolites biochemically linked to the affected gene (mPCVs), by retaining only gene-metabolite pairs reported as metabolite quantitative trait loci (mQTLs) in genome-wide association studies (GWASs). We assumed that mQTLs indicate a functional link between the gene and the metabolite.^20^ We identified 103 mPCVs for 65 metabolites associated with 59 variants (grouped in 88 variant-metabolite pairs, Table S5). The FDR for mPCVs was 25% (Figure S2D).

We assessed sensitivity, by varying the thresholds from the 20 most extreme value to the 10 most extreme, the five most extreme, and the mean ±3 standard deviations for both proteins and metabolites. The fractions of pPCVs (*in-cis* protein associations) and mPCVs (metabolite association reported in the mGWAS) reported in each dataset were similar, regardless of the threshold used (Figure S3). The similarity in the fractions of pPCV and mPCV suggests that more conservative thresholds do not exclude more false positive associations.

### Clinical outcome for 12 PCVs for which multiple data sources are available

We further dissected the potential effect of the association of PCVs with extreme protein and metabolite levels, by manually curating the pPCVs and mPCVs. We focus on 12 cases, 10 of which are the following: PCSK9, BHMT, ACY1, PLG, ACSM2A, ABCG5, ABCC2, PAOX, AFMID, and UPB1. We also included two other associations (AOX1 and ALOX15) that were reported in the Human Metabolite database (HMDB, Table 1). These PCVs are of particular interest, as they have (1) multiple sources of evidence, based on proteomics, metabolomics, and laboratory data, and/or (2) more than one homozygote in QBB displaying the extreme phenotype and therefore allow comparison of multiple cases for consistency.

**Table 1:** Twelve showcase pPCV and mPCV associations

Gene	Variant(s)	No. of homozygotes in QBB	Proteomics	Evidence	Laboratory data
				Metabolomics	
PCSK9	rs11591147 rs746442570	1 1	Low PCSK9	–	Low LDL-C
BHMT^a^ SLC6A12 CBS SLC6A5	chr5:78417119 chr12:301795 rs543307278 rs543307278	1 2 1 1	–	High betaine and differential dimethylglycine levels (high in A and B, low in C and D)	–
ACY1	rs121912698 rs2229152	1 1	Low ACY1	High acetylated amino acid levels	–
PLG	rs4252129	1	Low levels of plasminogen, angiostatin, and coagulation factors	–	High INR, prolonged PT, and prolonged APTT
ACSM2A	rs59261767 chr16:20480888	4 1	–	High indolepropionic acid and high phenylpropanoic acid levels	–
ABCG5	rs569748582 rs145164937	1 2	–	High campesterol and high beta-sitosterol levels	–
ABCC2	rs867979691 rs140680467	2 2	–	High bile-acid levels	–
PAOX	rs150446594	2	–	High spermidine-related metabolite levels	–
AFMID	rs77585764	4	–	High formylanthranilic acid levels	–
UPB1	rs138081800 rs145766755	2 1	–	High ß-ureidopropionate and low ß-aminoisobutyrate levels	–
ALOX15	rs41432647	1	–	High fatty-acid levels	–
AOX1	rs866541106	3	–	Low pyridoxate, high methylnicotinamide, and low N1-methyl-2-pyridone-5-carboxamide	–

A detailed vignette outlining all the available evidence for each showcase is provided as supplementary text.

APTT, activated partial thromboplastin time; INR, international normalized ratio; LDL-C, low-density lipoprotein cholesterol; PT, prothrombin time.

a High betaine levels were associated with PCVs in four genes (BHMT, SLC6A12, CBS, and SLC6A5).

As per comparison, we ran two types of rare variant association with metabolites and laboratory data analyses: (1) a burden test using the combined multivariate and collapsing method (CMC),^21^ and (2) the sequence kernel association test (SKAT).^22^ The burden test aggregates PCVs that impact the metabolite and the lab data levels in the same direction, whereas SKAT considers PCVs in opposite directions. We identified eight significant gene-laboratory phenotype associations and 59 significant gene-metabolite associations with the burden test. Using SKAT, we detected 14 gene-laboratory phenotype associations and 121 gene-metabolite associations (Table S6). Five of the 10 cases involving extreme metabolite levels (ACY1, ABCG5, ABCC2, PAOX, and UPB1) could also be identified in a burden test approach while the other five (genes associated with extreme betaine levels, ACSM2A, AFMID, ALOX15, and AOX1) were only found using the variant-extreme phenotype approach (Table S6). The two cases involving extreme laboratory data, PCSK9 and PLG, were also only found using the variant-extreme phenotype approach.

For two of the 12 cases—PCSK9 and PLG—extreme associations for proteomics and laboratory data were identified. For the ACY1 case, both protein (ACY1) and metabolite (acetylated amino acids) levels were extreme. For the betaine case, five homozygotes for PCVs of various genes (BHMT, SLC6A12, CBS, and SLC6A5) were associated with high betaine levels and different levels of dimethylglycine: high in individuals homozygous for the BHMT and SLC6A12 variants and low in individuals homozygous for the CBS and SLC6A5 variants. The eight remaining cases involved various homozygotes for a single gene that were found to be associated with one or several metabolites, such as ACSM2A with indolepropionic acid and phenylpropanoic acid; ABCC2 with the glycolic acid sulfate; PAOX with polyamine and polyamine metabolites, acetylspermidine, acisoga, and acetylisoputreanine; AFMID with formylanthranilic acid; UPB1 with beta-ureidopropionic acid and beta-aminoisobutyric acid; AOX1 with pyridoxic acid, methylnicotinamide, and N1-methyl-2-pyridone-5-carboxamide; and ALOX15 with linoleic acid and arachidonic acid.

We describe below three cases that are well-established in the literature (PCSK9, ACY1, and Plasmin). We discuss the nine other cases in detail in the Supplementary Text.

**Figure 4:**
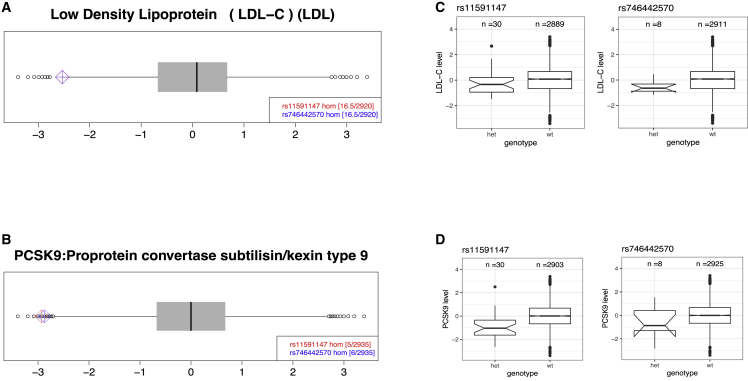
Two *PCSK9* missense variants cause low PCSK9 and LDL-C levels (A) LDL-C levels were similar and low in two individuals homozygous for missense variants (rs11591147 and rs746442570). (B) PCSK9 levels were extremely low for the two individuals homozygous for missense variants. (C) Overall LDL-C level for the PCSK9 heterozygotes was lower than that in wild-type individuals. (D) Overall PCSK9 level for the heterozygotes was lower than that in wild-type individuals. Het, heterozygous; wt, wild-type homozygous. All the protein and clinical biochemistry data are presented on plots with a normalized scale (*Z* score, mean = 0, SD = 1).

We identified one individual homozygous for the extensively studied PCSK9 missense variant rs11591147 in QBB. This variant has been reported to lower the levels of LDL in the blood and to reduce the risk of coronary heart disease. Confirming this finding, QBB homozygote for rs11591147 had a low level of LDL-C (Figure 4A). Our proteomics data also show that the homozygote of rs11591147 had lower levels of PCSK9 protein in the blood too (Figure 4B). The rs11591147 missense variant appears to have an additive effect on both PCSK9 (Figure 4C) and LDL-C levels (Figure 4D). We identified a second homozygous PCSK9 missense variant, rs746442570, carried by a single QBB participant. Unlike rs11591147, only two heterozygotes in ∼400,000 individuals were identified for rs746442570 in UKB.

**Figure 5:**
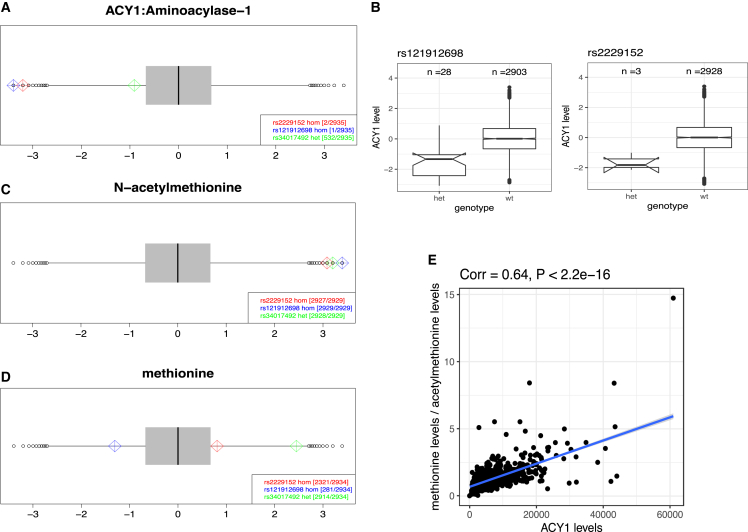
Two ACY1 missense variants cause low levels of ACY1 and high levels of N-acetylated amino acids (A) The ACY1 levels of two individuals homozygous for ACY1 variants (rs2229152 and rs121912698) were the lowest amount QBB participants. Another QBB participant carries a heterozygous ACY1 missense variant (rs34017492) and normal ACY1 levels. (B) Both rs2229152 and rs121912698 heterozygotes have lower ACY1 levels than wild-type individuals. (C) An example of high levels of an acetylated amino acid, acetylmethionine, in two ACY1 PCV homozygotes and one ACY1 PCV heterozygote. The data for the other acetylated amino acids determined are available in Table S7. (D) Example of free amino acid levels for methionine. Only the individual heterozygous for an ACY1 PCV had high methionine levels. The data for the other amino acids are presented in Table S7. (E) ACY1 levels were correlated with the ratio of methionine to acetylmethionine levels. All the protein and metabolite data are presented on plots with a normalized scale (*Z* score, mean = 0, SD = 1).

We identified two ACY1 missense variants, rs121912698 and rs2229152, each carried by a single homozygote in QBB. Both proteomics and metabolomics data provided evidence for an effect of these two missense variants of *ACY1* on blood circulating protein and metabolite levels: the levels of ACY1 protein in the rs121912698 and rs2229152 homozygotes were the lowest of the 2,935 QBB participants (Figure 5A). ACY1 is required to remove the acetyl group from acetylated amino acids. Therefore, in metabolomics analyses, the levels of various acetylated amino acids, including acetylmethionine, acetylalanine, acetylisoleucine, acetylleucine, acetylvaline, acetylglutamate, acetylhistidine, acetylserine, and acetylthreonine, were extremely high and ranked in the 20 highest levels in both rs121912698 and rs2229152 homozygotes (Figure 5C and Table S7). ACY1 deficiency is a neurological disorder caused by mutations in *ACY1* and characterized by high levels of acetylated amino acids. rs121912698 is an established causal variant of ACY1 deficiency^23^^,^^24^^,^^25^^,^^26^ where the replacement of Arg353 by a Cys residue could create a perturbation in the vicinity of the *ACY1* active site.^27^ Our data suggest that carriers of the rs2229152 variant are likely to present with the same pathophysiology. High levels of ACY1 expression are associated with a risk of type 2 diabetes (T2D).^28^^,^^29^^,^^30^ Free amino acid levels generated by overexpression of ACY1 play a role in insulin secretion and glucose homeostasis and could eventually lead to T2D with impaired ß-cell function and insulin resistance.^30^ Further investigations are, therefore, required to assess the potential of lowering free amino acid levels by ACY1 blockade as a potential treatment for T2D.

**Figure 6:**
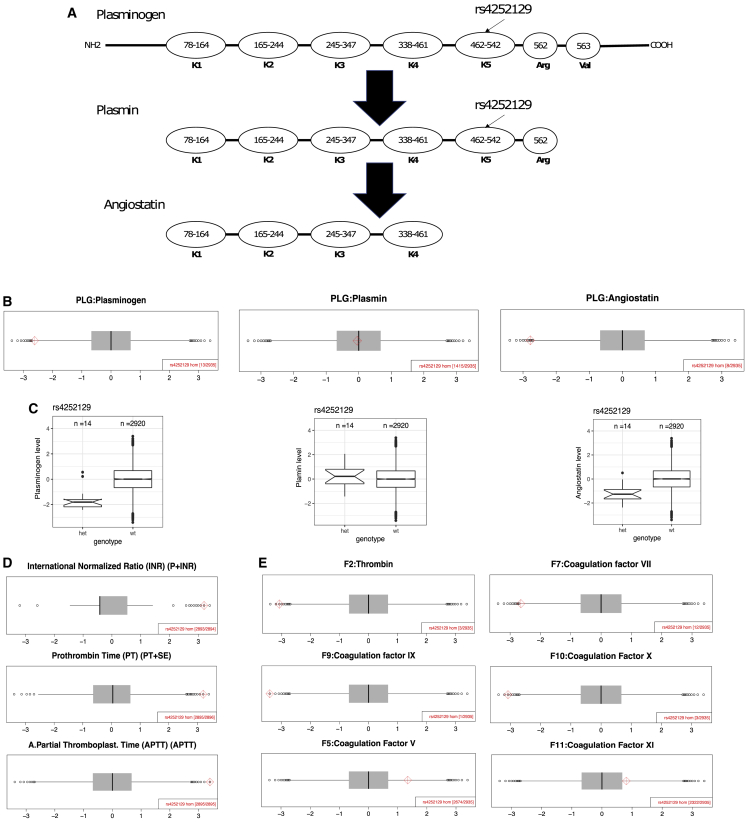
A homozygous carrier of the PLG missense mutation rs4252129 has low plasminogen and angiostatin levels but normal plasmin levels (A) Structure of the plasminogen, active plasmin, and angiostatin proteins. K1-K5 indicate the Kringle domains. (B) Plasminogen and angiostatin levels for the rs4252129 homozygote were low, whereas plasmin levels were normal. (C) rs4252129 heterozygotes have lower plasminogen and angiostatin levels than wild-type individuals, but similar plasmin levels. (D) Three independent tests measuring blood coagulation showed the measurements for the rs4252129 homozygote to be outliers. (E) The rs4252129 homozygote has low levels of four coagulation factors: F2, F7, F9, and F10 and normal levels for two other coagulation factors: F5 and F11. (B), (C), and (E) involved measurements on a SomaLogic platform, whereas (D) shows biochemical measurements. All the protein and clinical biochemistry data are presented on plots with a normalized scale (*Z* score, mean = 0, SD = 1).

We identified one homozygote for a PLG missense variant rs4252129 in QBB. The rs4252129 homozygote has low levels of plasminogen (plasmin zymogen) and angiostatin (the plasmin cleavage product) but normal level of active plasmin (Figure 6B). Individuals heterozygous for rs4252129 have lower levels of plasminogen and angiostatin but normal levels of active plasmin compared with the wild-type individuals, confirming the potential effect of rs4252129 on plasminogen and angiostatin rather than active plasmin (Figure 6C). Plasmin plays a role in fibrin clot degradation, and mutations in PLG were associated with severe thrombosis.^31^ The medical questionnaire completed by QBB participants indicated that the individual homozygous for rs4252129 was on warfarin treatment possibly for a high blood-clotting problem. This treatment inhibits the vitamin K-dependent synthesis of biologically active forms of the CFs F2, F7, F9, and F10. Our proteomics data determined the levels of six coagulation factors (CFs), four of which—F2, F7, F9, and F10—were extremely low in the rs4252129 homozygote (Figure 6E). Furthermore, our laboratory data showed that the rs4252129 homozygote has a prolonged activated partial thromboplastin time (APTT) and prothrombin time (PT) and a high international normalized ratio (INR) (Figure 6D). The low levels of four CFs, the prolonged APTT and PT, and high INR are in the rs4252129 homozygote probably a result of the warfarin treatment. The variant rs4252129 seems to extremely decrease both plasminogen and angiostatin levels in the blood, possibly resulting in a high blood-clotting problem. Further investigations are therefore required to determine whether extremely low levels of plasminogen and angiostatin combined with normal levels of active plasmin favor excessive blood clotting.

A detailed description of the three cases discussed here (PCSK9, ACY1, and PLG) and of the other nine cases is provided in the form of vignettes in the supplementary text.

## Discussion

We identified homozygous PCVs causing extreme protein and metabolite levels in QBB. Our data show that the population included in QBB is highly consanguineous.^32^ Almost half the PCVs identified in QBB have no homozygote in the largest available DNA sequencing catalog. Furthermore, most of the PCVs identified were detected in large ROH enriched in deleterious variants.^18^^,^^33^ In addition, previous studies to identify PCVs in cohorts well-characterized phenotypically used exome sequencing^7^^,^^34^ or imputed SNP arrays,^35^ but genome sequencing has been shown to outperform exome sequencing for identifying exonic variants^36^ and it is difficult to tag rare variants with the available imputation methods.^37^

In our extreme metabolite associations, we excluded individuals with undetected metabolite levels from the analysis. However, a PCV can disrupt the gene function related to the production of a specific metabolite resulting in undetected metabolite levels.^38^ Metabolites not detected in participants might therefore bias the mPCVs identified, making it impossible to determine whether the lack of detection is a random effect or due to an extreme level of the metabolite concerned. For undetected metabolite levels, we performed the Fisher test for randomness (STAR Methods) to detect whether the missing metabolite values from certain genotype groups were missing by chance. We also repeated the analysis using a value of zero for undetected metabolites, but no additional detections were achieved.

We identified PCVs causing extreme levels of proteins and metabolites, and extreme biochemical data, reflecting a disruption of gene function. The PCVs identified were PCSK9, BHMT, ACY1, PLG, ACSM2A, ABCG5, ABCC2, PAOX, AFMID, UPB1, AOX1, and ALOX15. PCSK9 provides proof-of-concept for our strategy for identifying true associations.^39^^,^^40^ Pharmacological inhibitors of PCSK9 lowering LDL-C levels and, thus, reducing the risk of coronary heart disease, were developed after the identification of healthy PCSK9 carriers, a process that could have been initiated by our analysis.

In summary, we report here evidence for both new and previously reported associations between genetic variants and extreme protein and metabolite levels in a consanguineous population. The protein (Table S2) and metabolite (Table S3) associations identified are presented in the supplementary tables. We believe that our findings will be of great utility for the screening of diseases and traits associated with the identified genes.^41^ Furthermore, this research should provide new hypotheses concerning potential targets for drug target investigations.

### Limitations of the study

Our study also has some limitations. First, genetic variation affecting the protein sequence may lead to changes in the higher order structure of the protein and, thus, its aptamer binding affinity.^42^ This may lead in some cases to extreme protein-level readouts where in reality only the aptamer binding affinity is affected by the variant. Additional pPCV findings concerning functional disruption in the metabolite and/or biochemical data can help to address this epitope effect. Second, many pPCVs and mPCVs, particularly those identified in a single participant, are false positives.^7^^,^^34^^,^^43^ We addressed this issue by manually curating the pPCVs and mPCVs. A larger sample size, such as the target size of QBB (60,000 participants), would also help to resolve this issue. Finally, the available proteomics and metabolomics platforms target a limited number of proteins and metabolites, respectively. The expected improvements to these platforms in the future will increase the number of proteins and metabolites that can be targeted.^44^^,^^45^

## Consortia

The members of the Qatar Genome Program Research Consortium are Said I. Ismail, Wadha Al-Muftah, Radja Badji, Hamdi Mbarek, Dima Darwish, Tasnim Fadl, Heba Yasin, Maryem Ennaifar, Rania Abdellatif, Fatima Alkuwari, Muhammad Alvi, Yasser Al-Sarraj, Chadi Saad, Asmaa Althani, Eleni Fethnou, Fatima Qafoud, Eiman Alkhayat, Nahla Afifi, Sara Tomei, Wei Liu, Stephan Lorenz, Najeeb Syed, Hakeem Almabrazi, Fazulur Rehaman Vempalli, Ramzi Temanni, Tariq Abu Saqri, Mohammedhusen Khatib, Mehshad Hamza, Tariq Abu Zaid, Ahmed El Khouly, Tushar Pathare, Shafeeq Poolat, Rashid Al-Ali, Omar Albagha, Souhaila Al-Khodor, Mashael Alshafai, Ramin Badii, Lotfi Chouchane, Xavier Estivill, Khalid Fakhro, Hamdi Mbarek, Younes Mokrab, Jithesh V. Puthen, Karsten Suhre, and Zohreh Tatari.

## STAR★Methods

### Key resources table

**Table:** 

REAGENT or RESOURCE	SOURCE	IDENTIFIER
**Other**		

QBB		https://www.qatarbiobank.org.qa
1,000 Genomes	1,000 Genomes Project Consortium et al., 2015^104^	https://www.internationalgenome.org
GnomAD	Karczewski et al., 2020^12^	https://gnomad.broadinstitute.org
UKB	Bycroft et al., 2018^105^	https://www.ukbiobank.ac.uk
GWAS catalog	MacArthur et al., 2017^106^	https://www.ebi.ac.uk/gwas/
HMDB	Wishart et al., 2018^107^	https://hmdb.ca
Drugbank		https://go.drugbank.com

**Software and algorithms**		

FastQC		https://www.bioinformatics.babraham.ac.uk/projects/fastqc/
BWA	Li et Durbin., 2010^108^	https://github.com/lh3/bwa/tree/master/bwakit
GATK	GATK team	https://gatk.broadinstitute.org/hc/en-us
Plink	Chang et al., 2015^109^	https://www.cog-genomics.org/plink/2.0/
VEP	McLaren et al., 2016^110^	https://useast.ensembl.org/info/docs/tools/vep/index.html
CADD	Kircher et al., 2014^111^	https://cadd.gs.washington.edu
REVEL	Ioannidis et al., 2016^112^	https://sites.google.com/site/revelgenomics/
bcftools	Narasimhan et al., 2016^113^	http://samtools.github.io/bcftools/bcftools.html
rvtests	Zhan et al., 2016^114^	http://zhanxw.github.io/rvtests/
Phenoscanner	Kamat et al., 2019^115^	http://www.phenoscanner.medschl.cam.ac.uk

### Resource availability

#### Lead contact

Further information and requests may be directed to the lead contact Karsten Suhre (kas2049@qatar-med.cornell.edu).

#### Materials availability

This study did not generate new unique reagents.

### Experimental model and subject details

#### Overview of the Qatar biobank (QBB)

QBB is a prospective, population-based cohort study established in 2012 and aims to eventually recruit 60,000 participants from Qatar.^14^^,^^15^ All the participants are adults and are either Qatari or have been resident in Qatar for at least 15 years. The participants included in this study signed a consent form before inclusion. The study was approved by the Hamad Medical Corporation Ethics Committee and QBB institutional review board. Associations of homozygous protein-changing variants with extreme protein and metabolite levels were analyzed for the first 2,935 participants included in QBB.

### Method details

#### Whole-genome sequencing

DNA was extracted from peripheral blood with the automated QIASymphony SP instrument, according to the manufacturer’s instructions (Qiagen, Germany). DNA was quantified in the Quant-iT dsDNA Assay (Invitrogen, USA) on a FlexStation 3 machine (Molecular Devices, USA). Whole-genome sequencing was performed with 2 × 150 bp paired-end reads on a HiSeq X Ten sequencer (Illumina, USA) at the Sidra Clinical Genomics Laboratory Sequencing Facility. The mean depth of coverage was 30x. Quality control was performed on the reads (Fastq files) with FastQC (v0.11.2) (https://www.bioinformatics.babraham.ac.uk/projects/fastqc/). Reads were then aligned with the GRCh37 (hs37d53) reference genome, with bwa.kit (v0.7.12) (https://github.com/lh3/bwa/tree/master/bwakit). Quality control was performed on the mapped reads with Picard (v1.117) [CollectWgsMetrics] (https://gatk.broadinstitute.org/hc/en-us). Variant calling was performed in accordance with GATK 3.4 best practices (https://software.broadinstitute.org/gatk/documentation/article?id=3238): indel realignment and base recalibration were performed on the initial bam file and HaplotypeCaller was then run on each sample to generate an intermediate genomic variant call file (gVCF). Joint variant calling was performed simultaneously on all the gVCF files generated. We first ran GenomicsDB8 to combine the different samples by region, and we then ran GenotypeGVCFs for each region. We applied the variant quality score recalibrator (VQSR) for SNVs and indels separately. In total, 105 million variants passed the VQSR filter for 2,935 QBB individuals.^116^ No individual was removed due to high rates of missing data (>0.1) with Plink (v2.0).^109^ Almost five million variants were removed due to a low calling rate (<0.05).

#### Variant annotation

The Variant Effect Predictor (VEP) was used to annotate the 100 million variants identified.^110^ The UTRannotator plugin was also used to annotate additional variants to create new upstream open reading frames (ORFs).^117^ In total, VEP annotated 610,493 autosomal variants as ORFs or as having a high or moderate impact.

Common functional alleles are less likely to exert strong functional effects as they are less constrained by purifying selection. We, thus, retained 35,567 protein-changing variants (PCVs) for which one to five homozygotes were present in QBB for analysis. To identify rare PCV that were not present in the non-consanguineous population, we excluded 2,699 PCVs with a minor allele frequency (MAF) ≥ 0.05 in the GnomAD populations. We finally retained 32,868 PCVs in the analysis and we annotated the identified PCVs with both the Combined Annotation Dependent Depletion (CADD)^111^ and the rare exome variant ensemble learner (REVEL)^112^ scores.

#### Estimation of the inbreeding coefficient F

The coefficient of inbreeding distributions of 2,935 QBB individuals were compared with those of 503 Europeans from the 1,000 genomes project.^104^ We extracted a total of 869,201 polymorphic SNPs present on the Affymetrix 6.0 SNP array that passed the quality control check-up in QBB and the 1,000 Genomes project (1KG). PLINK (v2.0) was used to estimate the coefficient of inbreeding separately for each ethnic group.^109^ The coefficient of inbreeding was estimated by dividing the observed degree of homozygosity by the expected homozygosity based on an estimated common ancestor.^118^ Two-sided Wilcoxon tests were used to compare the F coefficients of QBB population and the non-consanguineous 1KG population.

#### Runs of homozygosity (ROH)

We estimated the mean proportion of the genome covered by ROH in QBB participants, as previously described for the East London Genes & Health (ELGH) study^34^: a hidden Markov model implemented in bcftools^113^ was applied to the exonic regions targeted by the Illumina Trueseq exome regions (v1.2): bcftools roh -R exonic_regions_chr{CHROM).bed -G30 -a1e-8 -H1e-8 -V1e-10 -m genetic_map_chr{CHROM}_combined_b37.txt. We targeted exonic regions only, as ELGH is a collection of exome-sequencing data. Furthermore, ROH length does not differ significantly between exome- and genome-sequencing data obtained with bcftools.^113^ The proportion of the genome covered by ROH for each QBB participant was calculated with an in-house python script.

#### Expected homozygote frequency (EHF)

The EHF in the GnomAD consanguineous population was estimated as EHF = *(1 – a)p^2^ + ap*, where *p* is the cumulative allele frequency (CAF) estimated for all GnomAD individuals and *a* is the mean proportion of the genome covered by ROH in individuals self-reporting having second-cousin or more closely related parents in the ELGH study ^5419^. In GnomAD, the CAF was calculated as p = 1 – sqrt(*q*), where *q* is the fraction of GnomAD participants without loss of function. This calculation therefore requires the individual genotyping of GnomAD participants and is time-consuming. Here, we calculated the ‘classic’ CAF, as described by Minikel et al.^9^ in Extended Data Figure 3. The ‘classic’ CAF for each gene is the sum of allele frequencies for all rare variants (MAF <5%) annotated as “high-confidence loss-of-function” by Loftee^12^ in 125,748 GnomAD exomes.

We applied the method used on the GnomAD consanguineous population to estimate the EHF in QBB. The EHF for the GnomAD non-consanguineous population was estimated at four per hundred million for the median gene, a value different from the EHF reported by Minikel et al.^9^ (six per billion). This difference is probably due to the ‘classic’ CAF used here to estimate the EHF in the GnomAD non-consanguineous population.

#### PCVs associated with extreme protein and metabolite levels

The levels of 1,305 proteins were measured with the Somascan aptamer-based proteomics platform for 2,935 QBB participants. Six aptamers targeting human virus proteins were excluded. The Uniprot identifier for each protein was used in the BiomaRt package to identify the associated protein-coding genes.^119^ Fifty-eight aptamers were assigned to multiple Uniprot identifiers and included different protein isoforms and complexes. We treated each Uniprot identifier as a unique record, to avoid missing any interesting findings. In total, 1,301 unique protein-coding genes were linked to the 1,299 proteins analyzed.

For the identification of variants associated with extreme protein levels in QBB, we first browsed the 32,868 PCVs and identified homozygotes. For each PCV, there were one to five homozygotes. We restricted analyses to proteins for which all homozygotes ranked in the top 20 or the bottom 20 for protein levels. In total, 378,572 associations of 20,909 PCVs from 10,025 protein-coding genes with 1,301 proteins were identified.

The levels of 1,159 metabolites were determined for 2,935 QBB participants with the non-targeted HD4 metabolomics platform from Metabolon. For the identification of PCVs associated with extreme metabolite levels, we excluded participants with missing metabolite determinations. As for proteins, analyses of metabolite associations were restricted to situations in which all homozygotes ranked in the top 20 or the bottom 20 for metabolite levels. Only metabolites with non-missing measures for more than 1,000 QBB participants were kept. In total, we identified 312,899 associations of 29,999 PCVs from 11,967 protein-coding genes with 989 metabolites.

#### Estimation of the false discovery rate (FDR)

A knowledge of the distribution of a test statistic under the null hypothesis is important for FDR estimation. Permutation methods have become popular for estimating null distributions, due to their flexibility and generalizability. For both proteins and metabolites, we performed 100 permutations to estimate the null distribution. We randomly permuted the participant identifiers 100 times and determined the extreme associations for each permuted dataset, as for the unpermuted data. The FDR was estimated by dividing the mean number of associations in the permuted data by the number of associations identified in the unpermuted data.

#### Correction for multiple testing

We corrected for multiple testing and decreased the FDR for protein associations, by retaining associations for which the PCVs causing extreme protein level were variants of the gene encoding the protein concerned, that is, *in cis* associations (pPCVs).

For metabolites, we decreased the FDR by extracting pairs of genes and metabolites from metabolite associations already reported in the available mGWAS (mQTLs) (mPCVs). We extracted the mQTLs from the mGWAS reported in the GWAS catalog.^106^ mQTLs were fine mapped and manually curated to identify causal genes.^120^ mQTLs from 21 mGWAS in the GWAS catalog were fine mapped and manually curated to identify causal genes (Table S9). We also used the supplementary data from two recent mGWAS that were not included in the GWAS catalog.^120^^,^^121^

The XML version of the HMDB^107^ was downloaded from the HMDB website (https://hmdb.ca/) to identify metabolite associations reported in the HMDB. An in-house python script was used to extract metabolite-enzyme and metabolite-transporter associations. We linked the metabolites with a known HMDB identifier to enzymes, transporters and carriers as reported in the HMDB. The complete list of all associations is also provided in Table S9.

#### Fisher’s exact test for the randomness of missing values

Missing data can be classified in three patterns: missing completely at random (MCAR), missing at random (MAR), or missing not at random (MNAR). The missingness is MCAR, if the probability of being missing is the same for all cases; MAR, if the probability of being missing is the same only within groups defined by the observed data; MNAR, if the probability of being missing depends on both observed and non-observed quantities. The missingness in most of metabolites is due to the limits of detection (LOD).^122^ This missingness is assumed left-censoring, a variant of MNAR.

We used Fisher’s exact test to determine whether the missing metabolite or laboratory data values for homozygotes were missing by chance, in analyses of the effects of PCVs on the metabolite level and laboratory data. The two inputs for Fisher’s exact test were: (a) the number of missing values for each genotype group (wild-type, heterozygous and homozygous), and (b) the total number of samples in each genotype group. We used fisher.test method in R for this calculation. In case of missingness due to LOD, low *p* values indicate a low probability of these values being missing by chance.

#### Burden test for gene-metabolite associations

In gene-based association analyses, the effects of various variants on a gene unit are aggregated in burden and SKAT tests. We used the ‘FamCMC’ and ‘FamSKAT’ functions implemented in rvtests^114^ on the 610,493 autosomal variants as ORFs or as having a high or moderate impact. All 2,935 QBB participants were included, as both ‘FamCMC’ and ‘FamSKAT’ collapses and combines rare variants in related individuals through a kinship matrix. Variants with MAF >1% were excluded from the analysis. Sex, age, the three first principal components on genetics data were used as covariates. Metabolites and laboratory data with more than 300 missing values were excluded. The exome-wide significance was set at p < 2.39e-09 after Bonferroni correction for the maximum number of genes tested (18,798 genes) and the total number of phenotypes (71 laboratory phenotypes +1,040 metabolites).

#### Additional evidence for associations with extreme protein and metabolite levels

We collected additional evidence for pPCVs and mPCVs, by identifying homozygous participants. The extreme ranks for homozygotes were obtained for all phenotypes: 71 for laboratory data, 1,159 for metabolites and 1,299 for proteins. An approximate *p* value was calculated by dividing the product of extreme ranks for homozygotes by the total number of QBB participants to the power of the number of homozygotes:

$$P≅\frac{R_{1}}{N}*\ldots \frac{R_{k}}{N}$$

Where *k* is the number of homozygotes, *N* is the number of non-missing data points divided by 2 (division by 2 because we looked at both ends of the extreme) and *R*_*i*_ is the extreme rank of homozygote *i*.

After correction for multi-testing, a significant p-value will be:

$P<\frac{0.05}{M}$ Where *M* is the total number of phenotypes: *M =* 2,529 (71 laboratory data phenotypes +1,159 metabolites +1,299 proteins). A quantile-quantile plot (Figure S4) confirms that this p value is not inflated.

A comparison of the observed association statistics against the expected distribution suggests no systematic over-dispersion of the association statistics (Figure S4).

Note that the same extreme ranks for high and low levels give the same *P*. For example, if three homozygotes for one or many PCVs for the same gene have the levels of a specific phenotype ranked 2,935/2,935, 2,934/2.935 and 2,933/2,935, then the corresponding extreme ranks are 1/2,935, 2/2,935 and 3/2,935 and *P* = (1 ∗ 2 ∗ 3)/2,935^3^ = 2.37e-10. A low *p* value for a phenotype indicates a statistically significant association.

Genome-wide association study hits, expression quantitative trait loci (QTL), protein QTL, metabolite QTL, and methylation QTL displaying strong linkage disequilibrium with pPCVs and mPCVs in the European population (r^2^ > 0.8) were extracted with Phenoscanner.^115^

We used the R package VarfromPDB^123^ to identify diseases associated with pPCVs and mPCVs in Clinvar.^124^

We downloaded the DrugBank database (https://go.drugbank.com) and used the lxmx toolkit implemented in Python (v.2.7) to identify genes carrying pPCVs and mPCVs associated with drugs. We included only gene-drug combinations with a known action of the drugs on the genes.

### Quantification and statistical analysis

The quantitative and statistical analyses are described in the relevant sections of the method details or in the table and figure legends.

## Data Availability

All relevant data are provided in the Supplementary Data. Full access to QBB/QGP genotype and phenotype data can be obtained through an established ISO-certified process by submitting a project request to https://www.qatarbiobank.org.qa/research/how-apply, subject to approval by QBB IRB committee. Approved researchers can access UK Biobank data by applying at https://www.ukbiobank.ac.uk/enable-your-research/ apply-for-access. The 1,000 Genomes project, the Gnomad allele and genotype frequency, the GWAS catalog, the HMDB and the Drugbank data are publicly available and listed in the key resources table. This study did not generate original code.

## Supporting citations

The following references appear in the Supplemental information: ^46^^,^^47^^,^^48^^,^^49^^,^^50^^,^^51^^,^^52^^,^^53^^,^^54^^,^^55^^,^^56^^,^^57^^,^^58^^,^^59^^,^^60^^,^^61^^,^^62^^,^^63^^,^^64^^,^^65^^,^^66^^,^^67^^,^^68^^,^^69^^,^^70^^,^^71^^,^^72^^,^^73^^,^^74^^,^^75^^,^^76^^,^^77^^,^^78^^,^^79^^,^^80^^,^^81^^,^^82^^,^^83^^,^^84^^,^^85^^,^^86^^,^^87^^,^^88^^,^^89^^,^^90^^,^^91^^,^^92^^,^^93^^,^^94^^,^^95^^,^^96^^,^^97^^,^^98^^,^^99^^,^^100^^,^^101^^,^^102^^,^^103^

## Supplemental information

Document S1. Figures S1–S4 and Data S1

Table S1. List of 71 laboratory phenotypes assessed for QBB participants, related to STAR Methods

Table S2. All extreme protein-level variant associations, related to Figure 3

Table S3. All extreme metabolite-level variant associations, related to Figure 3

Table S4. *In-cis* extreme protein-level variant associations (pPCVs), related to Figure 3

Table S5. Extreme metabolite-level variant associations for the metabolite/gene pairs reported in the mGWAS, related to Figure 3

Table S6. Gene-wide significant burden associations with metabolites, for an MAF <1%, related to STAR Methods

Table S7. Associations of N-acetylated and free amino acid levels with two ACY1 missense variants, related to Table 1 and Figure 5

Table S8. Associations of lipid metabolites with the ALOX15 variant rs41432647, related to Table 1

Table S9. List of metabolite-gene associations from the mGWAS and the HMDB used to annotate the metabolite associations (mPCVs), related to Figure 3 and STAR Methods

Document S2. Article plus supplemental information

## References

[1] Zhao Z., Tuakli-Wosornu Y., Lagace T.A., Kinch L., Grishin N.V., Horton J.D., Cohen J.C., Hobbs H.H. (2006). Molecular characterization of loss-of-function mutations in PCSK9 and identification of a compound heterozygote. Am. J. Hum. Genet..

[2] Schwartz G.G., Steg P.G., Szarek M., Bhatt D.L., Bittner V.A., Diaz R., Edelberg J.M., Goodman S.G., Hanotin C., Harrington R.A. (2018). Alirocumab and cardiovascular outcomes after acute coronary syndrome. N. Engl. J. Med..

[3] Sabatine M.S., Giugliano R.P., Keech A.C., Honarpour N., Wiviott S.D., Murphy S.A., Kuder J.F., Wang H., Liu T., Wasserman S.M. (2017). Evolocumab and clinical outcomes in patients with cardiovascular disease. N. Engl. J. Med..

[4] Cohen J., Pertsemlidis A., Kotowski I.K., Graham R., Garcia C.K., Hobbs H.H. (2005). Low LDL cholesterol in individuals of African descent resulting from frequent nonsense mutations in PCSK9. Nat. Genet..

[5] Mullard A. (2017). Calls grow to tap the gold mine of human genetic knockouts. Nat. Rev. Drug Discov..

[6] Lim E.T., Würtz P., Havulinna A.S., Palta P., Tukiainen T., Rehnström K., Esko T., Mägi R., Inouye M., Lappalainen T. (2014). Distribution and medical impact of loss-of-function variants in the Finnish founder population. PLoS Genet..

[7] Saleheen D., Natarajan P., Armean I.M., Zhao W., Rasheed A., Khetarpal S.A., Won H.-H., Karczewski K.J., O’Donnell-Luria A.H., Samocha K.E. (2017). Human knockouts and phenotypic analysis in a cohort with a high rate of consanguinity. Nature.

[8] McGregor T.L., Hunt K.A., Yee E., Mason D., Nioi P., Ticau S., Pelosi M., Loken P.R., Finer S., Lawlor D.A. (2020). Characterising a healthy adult with a rare HAO1 knockout to support a therapeutic strategy for primary hyperoxaluria. Elife.

[9] Minikel E.V., Karczewski K.J., Martin H.C., Cummings B.B., Whiffin N., Rhodes D., Alföldi J., Trembath R.C., van Heel D.A., Daly M.J. (2020). Evaluating drug targets through human loss-of-function genetic variation. Nature.

[10] Szustakowski J.D., Balasubramanian S., Kvikstad E., Khalid S., Bronson P.G., Sasson A., Wong E., Liu D., Wade Davis J., Haefliger C. (2021). Advancing human genetics research and drug discovery through exome sequencing of the UK Biobank. Nat. Genet..

[11] MacArthur D.G., Balasubramanian S., Frankish A., Huang N., Morris J., Walter K., Jostins L., Habegger L., Pickrell J.K., Montgomery S.B. (2012). A systematic survey of loss-of-function variants in human protein-coding genes. Science.

[12] Karczewski K.J., Francioli L.C., Tiao G., Cummings B.B., Alföldi J., Wang Q., Collins R.L., Laricchia K.M., Ganna A., Birnbaum D.P. (2020). The mutational constraint spectrum quantified from variation in 141, 456 humans. Nature.

[13] Lek M., Karczewski K.J., Minikel E.V., Samocha K.E., Banks E., Fennell T., O’Donnell-Luria A.H., Ware J.S., Hill A.J., Cummings B.B. (2016). Analysis of protein-coding genetic variation in 60, 706 humans. Nature.

[14] Al Thani A., Fthenou E., Paparrodopoulos S., Al Marri A., Shi Z., Qafoud F., Afifi N. (2019). Qatar biobank cohort study: study design and first results. Am. J. Epidemiol..

[15] Mbarek H., Devadoss Gandhi G., Selvaraj S., Al-Muftah W., Badji R., Al-Sarraj Y., Saad C., Darwish D., Alvi M., Fadl T. (2022). Qatar genome: insights on genomics from the Middle East. Hum. Mutat..

[16] Kathiresan S., Myocardial Infarction Genetics Consortium (2008). A PCSK9 missense variant associated with a reduced risk of early-onset myocardial infarction. N. Engl. J. Med..

[17] Szpiech Z.A., Xu J., Pemberton T.J., Peng W., Zöllner S., Rosenberg N.A., Li J.Z. (2013). Long runs of homozygosity are enriched for deleterious variation. Am. J. Hum. Genet..

[18] Szpiech Z.A., Mak A.C.Y., White M.J., Hu D., Eng C., Burchard E.G., Hernandez R.D. (2019). Ancestry-dependent enrichment of deleterious homozygotes in runs of homozygosity. Am. J. Hum. Genet..

[19] Finer S., Martin H.C., Khan A., Hunt K.A., MacLaughlin B., Ahmed Z., Ashcroft R., Durham C., MacArthur D.G., McCarthy M.I. (2020). Cohort Profile: East London Genes & Health (ELGH), a community-based population genomics and health study in British Bangladeshi and British Pakistani people. Int. J. Epidemiol..

[20] Stacey D., Fauman E.B., Ziemek D., Sun B.B., Harshfield E.L., Wood A.M., Butterworth A.S., Suhre K., Paul D.S. (2019). ProGeM: a framework for the prioritization of candidate causal genes at molecular quantitative trait loci. Nucleic Acids Res..

[21] Li B., Leal S.M. (2008). Methods for detecting associations with rare variants for common diseases: application to analysis of sequence data. Am. J. Hum. Genet..

[22] Wu M.C., Lee S., Cai T., Li Y., Boehnke M., Lin X. (2011). Rare-variant association testing for sequencing data with the sequence kernel association test. Am. J. Hum. Genet..

[23] Van Coster R.N., Gerlo E.A., Giardina T.G., Engelke U.F., Smet J.E., De Praeter C.M., Meersschaut V.A., De Meirleir L.J., Seneca S.H., Devreese B. (2005). Aminoacylase I deficiency: a novel inborn error of metabolism. Biochem. Biophys. Res. Commun..

[24] Sass J.O., Mohr V., Olbrich H., Engelke U., Horvath J., Fliegauf M., Loges N.T., Schweitzer-Krantz S., Moebus R., Weiler P. (2006). Mutations in ACY1, the gene encoding aminoacylase 1, cause a novel inborn error of metabolism. Am. J. Hum. Genet..

[25] Sass J.O., Vaithilingam J., Gemperle-Britschgi C., Delnooz C.C.S., Kluijtmans L.A.J., van de Warrenburg B.P.C., Wevers R.A. (2016). Expanding the phenotype in aminoacylase 1 (ACY1) deficiency: characterization of the molecular defect in a 63-year-old woman with generalized dystonia. Metab. Brain Dis..

[26] Michelucci R., Mecarelli O., Bovo G., Bisulli F., Testoni S., Striano P., Striano S., Tinuper P., Nobile C. (2007). A de novo LGI1 mutation causing idiopathic partial epilepsy with telephone-induced seizures. Neurology.

[27] D’Ambrosio C., Talamo F., Vitale R.M., Amodeo P., Tell G., Ferrara L., Scaloni A. (2003). Probing the dimeric structure of porcine aminoacylase 1 by mass spectrometric and modeling procedures. Biochemistry.

[28] Elhadad M.A., Jonasson C., Huth C., Wilson R., Gieger C., Matias P., Grallert H., Graumann J., Gailus-Durner V., Rathmann W. (2020). Deciphering the plasma proteome of type 2 diabetes. Diabetes.

[29] Gudmundsdottir V., Zaghlool S.B., Emilsson V., Aspelund T., Ilkov M., Gudmundsson E.F., Jonsson S.M., Zilhão N.R., Lamb J.R., Suhre K. (2020). Circulating protein signatures and causal candidates for type 2 diabetes. Diabetes.

[30] Ngo D., Benson M.D., Long J.Z., Chen Z.-Z., Wang R., Nath A.K., Keyes M.J., Shen D., Sinha S., Kuhn E. (2021). Proteomic profiling reveals biomarkers and pathways in type 2 diabetes risk. JCI Insight.

[31] Bugge T.H., Flick M.J., Daugherty C.C., Degen J.L. (1995). Plasminogen deficiency causes severe thrombosis but is compatible with development and reproduction. Genes Dev..

[32] Razali R.M., Rodriguez-Flores J., Ghorbani M., Naeem H., Aamer W., Aliyev E., Jubran A., Clark A.G., Fakhro K.A., Mokrab Y., Qatar Genome Program Research Consortium (2021). Thousands of Qatari genomes inform human migration history and improve imputation of Arab haplotypes. Nat. Commun..

[33] Pemberton T.J., Absher D., Feldman M.W., Myers R.M., Rosenberg N.A., Li J.Z. (2012). Genomic patterns of homozygosity in worldwide human populations. Am. J. Hum. Genet..

[34] Narasimhan V.M., Hunt K.A., Mason D., Baker C.L., Karczewski K.J., Barnes M.R., Barnett A.H., Bates C., Bellary S., Bockett N.A. (2016). Health and population effects of rare gene knockouts in adult humans with related parents. Science.

[35] Cheng Y., Schlosser P., Hertel J., Sekula P., Oefner P.J., Spiekerkoetter U., Mielke J., Freitag D.F., Schmidts M., GCKD Investigators (2021). Rare genetic variants affecting urine metabolite levels link population variation to inborn errors of metabolism. Nat. Commun..

[36] Belkadi A., Bolze A., Itan Y., Cobat A., Vincent Q.B., Antipenko A., Shang L., Boisson B., Casanova J.-L., Abel L. (2015). Whole-genome sequencing is more powerful than whole-exome sequencing for detecting exome variants. Proc. Natl. Acad. Sci. USA.

[37] Marchini J., Howie B., Myers S., McVean G., Donnelly P. (2007). A new multipoint method for genome-wide association studies by imputation of genotypes. Nat. Genet..

[38] Yousri N.A., Fakhro K.A., Robay A., Rodriguez-Flores J.L., Mohney R.P., Zeriri H., Odeh T., Kader S.A., Aldous E.K., Thareja G. (2018). Whole-exome sequencing identifies common and rare variant metabolic QTLs in a Middle Eastern population. Nat. Commun..

[39] Cohen J.C., Boerwinkle E., Mosley T.H., Hobbs H.H. (2006). Sequence variations in PCSK9, low LDL, and protection against coronary heart disease. N. Engl. J. Med..

[40] Kent S.T., Rosenson R.S., Avery C.L., Chen Y.-D.I., Correa A., Cummings S.R., Cupples L.A., Cushman M., Evans D.S., Gudnason V. (2017). PCSK9 loss-of-function variants, low-density lipoprotein cholesterol, and risk of coronary heart disease and stroke: data from 9 studies of blacks and whites. Circ. Cardiovasc. Genet..

[41] Taliun D., Harris D.N., Kessler M.D., Carlson J., Szpiech Z.A., Torres R., Taliun S.A.G., Corvelo A., Gogarten S.M., Kang H.M. (2021). Sequencing of 53, 831 diverse genomes from the NHLBI TOPMed Program. Nature.

[42] Suhre K., Arnold M., Bhagwat A.M., Cotton R.J., Engelke R., Raffler J., Sarwath H., Thareja G., Wahl A., DeLisle R.K. (2017). Connecting genetic risk to disease end points through the human blood plasma proteome. Nat. Commun..

[43] Sulem P., Helgason H., Oddson A., Stefansson H., Gudjonsson S.A., Zink F., Hjartarson E., Sigurdsson G.T., Jonasdottir A., Jonasdottir A. (2015). Identification of a large set of rare complete human knockouts. Nat. Genet..

[44] Petrera A., von Toerne C., Behler J., Huth C., Thorand B., Hilgendorff A., Hauck S.M. (2021). Multiplatform approach for plasma proteomics: complementarity of olink proximity extension assay technology to mass spectrometry-based protein profiling. J. Proteome Res..

[45] Marx V. (2020). Boost that metabolomic confidence. Nat. Methods.

[46] Kathiresan S., Melander O., Guiducci C., Surti A., Burtt N.P., Rieder M.J., Cooper G.M., Roos C., Voight B.F., Havulinna A.S. (2008). Six new loci associated with blood low-density lipoprotein cholesterol, high-density lipoprotein cholesterol or triglycerides in humans. Nat. Genet..

[47] Qiu C., Zeng P., Li X., Zhang Z., Pan B., Peng Z.Y.F., Li Y., Ma Y., Leng Y., Chen R. (2017). What is the impact of PCSK9 rs505151 and rs11591147 polymorphisms on serum lipids level and cardiovascular risk: a meta-analysis. Lipids Health Dis..

[48] Verbeek R., Boyer M., Boekholdt S.M., Hovingh G.K., Kastelein J.J.P., Wareham N., Khaw K.-T., Arsenault B.J. (2017). Carriers of the PCSK9 R46L variant are characterized by an antiatherogenic lipoprotein profile Assessed by nuclear magnetic resonance spectroscopy-brief report. Arterioscler. Thromb. Vasc. Biol..

[49] Rao A.S., Lindholm D., Rivas M.A., Knowles J.W., Montgomery S.B., Ingelsson E. (2018). Large-scale phenome-wide association study of PCSK9 variants demonstrates protection against ischemic stroke. Circ. Genom. Precis. Med..

[50] Lu X., Peloso G.M., Liu D.J., Wu Y., Zhang H., Zhou W., Li J., Tang C.S.-M., Dorajoo R., Li H. (2017). Exome chip meta-analysis identifies novel loci and East Asian-specific coding variants that contribute to lipid levels and coronary artery disease. Nat. Genet..

[51] de Franchis R., Kraus E., Kozich V., Sebastio G., Kraus J.P. (1999). Four novel mutations in the cystathionine beta-synthase gene: effect of a second linked mutation on the severity of the homocystinuric phenotype. Hum. Mutat..

[52] Lee S.-J., Lee D.H., Yoo H.-W., Koo S.K., Park E.-S., Park J.-W., Lim H.G., Jung S.-C. (2005). Identification and functional analysis of cystathionine beta-synthase gene mutations in patients with homocystinuria. J. Hum. Genet..

[53] El-Said M.F., Badii R., Bessisso M.S., Shahbek N., El-Ali M.G., El-Marikhie M., El-Zyoid M., Salem M.S.Z., Bener A., Hoffmann G.F., Zschocke J. (2006). A common mutation in the CBS gene explains a high incidence of homocystinuria in the Qatari population. Hum. Mutat..

[54] Zschocke J., Kebbewar M., Gan-Schreier H., Fischer C., Fang-Hoffmann J., Wilrich J., Abdoh G., Ben-Omran T., Shahbek N., Lindner M. (2009). Molecular neonatal screening for homocystinuria in the Qatari population. Hum. Mutat..

[55] Ageno W., Gallus A.S., Wittkowsky A., Crowther M., Hylek E.M., Palareti G. (2012). Oral anticoagulant therapy: antithrombotic therapy and prevention of thrombosis, 9th ed: American College of chest physicians evidence-based clinical practice guidelines. Chest.

[56] Gately S., Twardowski P., Stack M.S., Cundiff D.L., Grella D., Castellino F.J., Enghild J., Kwaan H.C., Lee F., Kramer R.A. (1997). The mechanism of cancer-mediated conversion of plasminogen to the angiogenesis inhibitor angiostatin. Proc. Natl. Acad. Sci. USA.

[57] Dodd D., Spitzer M.H., Van Treuren W., Merrill B.D., Hryckowian A.J., Higginbottom S.K., Le A., Cowan T.M., Nolan G.P., Fischbach M.A., Sonnenburg J.L. (2017). A gut bacterial pathway metabolizes aromatic amino acids into nine circulating metabolites. Nature.

[58] Elsden S.R., Hilton M.G., Waller J.M. (1976). The end products of the metabolism of aromatic amino acids by Clostridia. Arch. Microbiol..

[59] Masters C.L., Simms G., Weinman N.A., Multhaup G., McDonald B.L., Beyreuther K. (1985). Amyloid plaque core protein in Alzheimer disease and Down syndrome. Proc. Natl. Acad. Sci. USA.

[60] Bendheim P.E., Poeggeler B., Neria E., Ziv V., Pappolla M.A., Chain D.G. (2002). Development of indole-3-propionic acid (OXIGON) for Alzheimer’s disease. J. Mol. Neurosci..

[61] Chyan Y.J., Poeggeler B., Omar R.A., Chain D.G., Frangione B., Ghiso J., Pappolla M.A. (1999). Potent neuroprotective properties against the Alzheimer beta-amyloid by an endogenous melatonin-related indole structure, indole-3-propionic acid. J. Biol. Chem..

[62] Karbownik M., Stasiak M., Zygmunt A., Zasada K., Lewiński A. (2006). Protective effects of melatonin and indole-3-propionic acid against lipid peroxidation, caused by potassium bromate in the rat kidney. Cell Biochem. Funct..

[63] Venkatesh M., Mukherjee S., Wang H., Li H., Sun K., Benechet A.P., Qiu Z., Maher L., Redinbo M.R., Phillips R.S. (2014). Symbiotic bacterial metabolites regulate gastrointestinal barrier function via the xenobiotic sensor PXR and Toll-like receptor 4. Immunity.

[64] Zhao Z.-H., Xin F.-Z., Xue Y., Hu Z., Han Y., Ma F., Zhou D., Liu X.-L., Cui A., Liu Z. (2019). Indole-3-propionic acid inhibits gut dysbiosis and endotoxin leakage to attenuate steatohepatitis in rats. Exp. Mol. Med..

[65] de Mello V.D., Paananen J., Lindström J., Lankinen M.A., Shi L., Kuusisto J., Pihlajamäki J., Auriola S., Lehtonen M., Rolandsson O. (2017). Indolepropionic acid and novel lipid metabolites are associated with a lower risk of type 2 diabetes in the Finnish Diabetes Prevention Study. Sci. Rep..

[66] Tuomainen M., Lindström J., Lehtonen M., Auriola S., Pihlajamäki J., Peltonen M., Tuomilehto J., Uusitupa M., de Mello V.D., Hanhineva K. (2018). Associations of serum indolepropionic acid, a gut microbiota metabolite, with type 2 diabetes and low-grade inflammation in high-risk individuals. Nutr. Diabetes.

[67] Connor W.E., Lin D.S., Pappu A.S., Frohlich J., Gerhard G. (2005). Dietary sitostanol and campestanol: accumulation in the blood of humans with sitosterolemia and xanthomatosis and in rat tissues. Lipids.

[68] Williams K., Segard A., Graf G.A. (2021). Sitosterolemia: twenty years of discovery of the function of ABCG5ABCG8. Int. J. Mol. Sci..

[69] Wang H.H., Liu M., Portincasa P., Wang D.Q.-H. (2020). Recent advances in the critical role of the sterol efflux transporters ABCG5/G8 in health and disease. Adv. Exp. Med. Biol..

[70] Portincasa P., Di Ciaula A., de Bari O., Garruti G., Palmieri V.O., Wang D.Q.-H. (2016). Management of gallstones and its related complications. Expet Rev. Gastroenterol. Hepatol..

[71] Kajinami K., Brousseau M.E., Nartsupha C., Ordovas J.M., Schaefer E.J. (2004). ATP binding cassette transporter G5 and G8 genotypes and plasma lipoprotein levels before and after treatment with atorvastatin. J. Lipid Res..

[72] Gylling H., Hallikainen M., Pihlajamäki J., Agren J., Laakso M., Rajaratnam R.A., Rauramaa R., Miettinen T.A. (2004). Polymorphisms in the ABCG5 and ABCG8 genes associate with cholesterol absorption and insulin sensitivity. J. Lipid Res..

[73] Berge K.E., von Bergmann K., Lutjohann D., Guerra R., Grundy S.M., Hobbs H.H., Cohen J.C. (2002). Heritability of plasma noncholesterol sterols and relationship to DNA sequence polymorphism in ABCG5 and ABCG8. J. Lipid Res..

[74] Kuo K.-K., Shin S.-J., Chen Z.-C., Yang Y.-H.C., Yang J.-F., Hsiao P.-J. (2008). Significant association of ABCG5 604Q and ABCG8 D19H polymorphisms with gallstone disease. Br. J. Surg..

[75] Katsika D., Magnusson P., Krawczyk M., Grünhage F., Lichtenstein P., Einarsson C., Lammert F., Marschall H.-U. (2010). Gallstone disease in Swedish twins: risk is associated with ABCG8 D19H genotype. J. Intern. Med..

[76] Yu L., Hammer R.E., Li-Hawkins J., Von Bergmann K., Lutjohann D., Cohen J.C., Hobbs H.H. (2002). Disruption of Abcg5 and Abcg8 in mice reveals their crucial role in biliary cholesterol secretion. Proc. Natl. Acad. Sci. USA.

[77] Alonso A., Yu B., Qureshi W.T., Grams M.E., Selvin E., Soliman E.Z., Loehr L.R., Chen L.Y., Agarwal S.K., Alexander D., Boerwinkle E. (2015). Metabolomics and incidence of atrial fibrillation in african Americans: the atherosclerosis risk in communities (ARIC) study. PLoS One.

[78] Gimenez F., Fernandez C., Mabondzo A. (2004). Transport of HIV protease inhibitors through the blood-brain barrier and interactions with the efflux proteins, P-glycoprotein and multidrug resistance proteins. J. Acquir. Immune Defic. Syndr..

[79] Weiss J., Theile D., Ketabi-Kiyanvash N., Lindenmaier H., Haefeli W.E. (2007). Inhibition of MRP1/ABCC1, MRP2/ABCC2, and MRP3/ABCC3 by nucleoside, nucleotide, and non-nucleoside reverse transcriptase inhibitors. Drug Metab. Dispos..

[80] Vujcic S., Liang P., Diegelman P., Kramer D.L., Porter C.W. (2003). Genomic identification and biochemical characterization of the mammalian polyamine oxidase involved in polyamine back-conversion. Biochem. J..

[81] Hugill A.J., Stewart M.E., Yon M.A., Probert F., Cox I.J., Hough T.A., Scudamore C.L., Bentley L., Wall G., Wells S.E., Cox R.D. (2015). Loss of arylformamidase with reduced thymidine kinase expression leads to impaired glucose tolerance. Biol. Open.

[82] Moolenaar S.H., Göhlich-Ratmann G., Engelke U.F., Spraul M., Humpfer E., Dvortsak P., Voit T., Hoffmann G.F., Bräutigam C., van Kuilenburg A.B. (2001). beta-Ureidopropionase deficiency: a novel inborn error of metabolism discovered using NMR spectroscopy on urine. Magn. Reson. Med..

[83] Kölker S., Okun J.G., Hörster F., Assmann B., Ahlemeyer B., Kohlmüller D., Exner-Camps S., Mayatepek E., Krieglstein J., Hoffmann G.F. (2001). 3-Ureidopropionate contributes to the neuropathology of 3-ureidopropionase deficiency and severe propionic aciduria: a hypothesis. J. Neurosci. Res..

[84] van Kuilenburg A.B.P., Meinsma R., Beke E., Assmann B., Ribes A., Lorente I., Busch R., Mayatepek E., Abeling N.G.G.M., van Cruchten A. (2004). beta-Ureidopropionase deficiency: an inborn error of pyrimidine degradation associated with neurological abnormalities. Hum. Mol. Genet..

[85] van Kuilenburg A.B.P., Dobritzsch D., Meijer J., Krumpel M., Selim L.A., Rashed M.S., Assmann B., Meinsma R., Lohkamp B., Ito T. (2012). ß-ureidopropionase deficiency: phenotype, genotype and protein structural consequences in 16 patients. Biochim. Biophys. Acta.

[86] Pryde D.C., Dalvie D., Hu Q., Jones P., Obach R.S., Tran T.-D. (2010). Aldehyde oxidase: an enzyme of emerging importance in drug discovery. J. Med. Chem..

[87] Smith M.A., Marinaki A.M., Arenas M., Shobowale-Bakre M., Lewis C.M., Ansari A., Duley J., Sanderson J.D. (2009). Novel pharmacogenetic markers for treatment outcome in azathioprine-treated inflammatory bowel disease. Aliment. Pharmacol. Ther..

[88] Hartmann T., Terao M., Garattini E., Teutloff C., Alfaro J.F., Jones J.P., Leimkühler S. (2012). The impact of single nucleotide polymorphisms on human aldehyde oxidase. Drug Metab. Dispos..

[89] Foti A., Hartmann T., Coelho C., Santos-Silva T., Romão M.J., Leimkühler S. (2016). Optimization of the expression of human aldehyde oxidase for investigations of single-nucleotide polymorphisms. Drug Metab. Dispos..

[90] Foti A., Dorendorf F., Leimkühler S. (2017). A single nucleotide polymorphism causes enhanced radical oxygen species production by human aldehyde oxidase. PLoS One.

[91] Coelho C., Muthukumaran J., Santos-Silva T., João Romão M. (2019). Systematic exploration of predicted destabilizing nonsynonymous single nucleotide polymorphisms (nsSNPs) of human aldehyde oxidase: a Bio-informatics study. Pharmacol. Res. Perspect..

[92] Torres R.A., Korzekwa K.R., McMasters D.R., Fandozzi C.M., Jones J.P. (2007). Use of density functional calculations to predict the regioselectivity of drugs and molecules metabolized by aldehyde oxidase. J. Med. Chem..

[93] Feltenmark S., Gautam N., Brunnström A., Griffiths W., Backman L., Edenius C., Lindbom L., Björkholm M., Claesson H.-E. (2008). Eoxins are proinflammatory arachidonic acid metabolites produced via the 15-lipoxygenase-1 pathway in human eosinophils and mast cells. Proc. Natl. Acad. Sci. USA.

[94] Claesson H.-E. (2009). On the biosynthesis and biological role of eoxins and 15-lipoxygenase-1 in airway inflammation and Hodgkin lymphoma. Prostag. Other Lipid Mediat..

[95] Assimes T.L., Knowles J.W., Priest J.R., Basu A., Borchert A., Volcik K.A., Grove M.L., Tabor H.K., Southwick A., Tabibiazar R. (2008). A near null variant of 12/15-LOX encoded by a novel SNP in ALOX15 and the risk of coronary artery disease. Atherosclerosis.

[96] Schurmann K., Anton M., Ivanov I., Richter C., Kuhn H., Walther M. (2011). Molecular basis for the reduced catalytic activity of the naturally occurring T560M mutant of human 12/15-lipoxygenase that has been implicated in coronary artery disease. J. Biol. Chem..

[97] Astle W.J., Elding H., Jiang T., Allen D., Ruklisa D., Mann A.L., Mead D., Bouman H., Riveros-Mckay F., Kostadima M.A. (2016). The allelic landscape of human blood cell trait variation and links to common complex disease. Cell.

[98] Kristjansson R.P., Benonisdottir S., Davidsson O.B., Oddsson A., Tragante V., Sigurdsson J.K., Stefansdottir L., Jonsson S., Jensson B.O., Arthur J.G. (2019). A loss-of-function variant in ALOX15 protects against nasal polyps and chronic rhinosinusitis. Nat. Genet..

[99] Barnig C., Levy B.D. (2015). Innate immunity is a key factor for the resolution of inflammation in asthma. Eur. Respir. Rev..

[100] Rogerio A.P., Haworth O., Croze R., Oh S.F., Uddin M., Carlo T., Pfeffer M.A., Priluck R., Serhan C.N., Levy B.D. (2012). Resolvin D1 and aspirin-triggered resolvin D1 promote resolution of allergic airways responses. J. Immunol..

[101] Cole B.K., Lieb D.C., Dobrian A.D., Nadler J.L. (2013). 12- and 15-lipoxygenases in adipose tissue inflammation. Prostag. Other Lipid Mediat..

[102] Qu Q., Xuan W., Fan G.-H. (2015). Roles of resolvins in the resolution of acute inflammation. Cell Biol. Int..

[103] Heras-Sandoval D., Pedraza-Chaverri J., Pérez-Rojas J.M. (2016). Role of docosahexaenoic acid in the modulation of glial cells in Alzheimer’s disease. J. Neuroinflammation.

[104] Auton A., Brooks L.D., Durbin R.M., Garrison E.P., Kang H.M., Korbel J.O., Marchini J.L., McCarthy S., McVean G.A., Abecasis G.R., 1000 Genomes Project Consortium (2015). A global reference for human genetic variation. Nature.

[105] Bycroft C., Freeman C., Petkova D., Band G., Elliott L.T., Sharp K., Motyer A., Vukcevic D., Delaneau O., O’Connell J. (2018). The UK Biobank resource with deep phenotyping and genomic data. Nature.

[106] MacArthur J., Bowler E., Cerezo M., Gil L., Hall P., Hastings E., Junkins H., McMahon A., Milano A., Morales J. (2017). The new NHGRI-EBI Catalog of published genome-wide association studies (GWAS Catalog). Nucleic Acids Res..

[107] Wishart D.S., Feunang Y.D., Marcu A., Guo A.C., Liang K., Vázquez-Fresno R., Sajed T., Johnson D., Li C., Karu N. (2018). Hmdb 4.0: the human metabolome database for 2018. Nucleic Acids Res..

[108] Li H., Durbin R. (2010). Fast and accurate long-read alignment with Burrows-Wheeler transform. Bioinformatics.

[109] Chang C.C., Chow C.C., Tellier L.C., Vattikuti S., Purcell S.M., Lee J.J. (2015). Second-generation PLINK: rising to the challenge of larger and richer datasets. GigaScience.

[110] McLaren W., Gil L., Hunt S.E., Riat H.S., Ritchie G.R.S., Thormann A., Flicek P., Cunningham F. (2016). The ensembl variant effect predictor. Genome Biol..

[111] Kircher M., Witten D.M., Jain P., O’Roak B.J., Cooper G.M., Shendure J. (2014). A general framework for estimating the relative pathogenicity of human genetic variants. Nat. Genet..

[112] Ioannidis N.M., Rothstein J.H., Pejaver V., Middha S., McDonnell S.K., Baheti S., Musolf A., Li Q., Holzinger E., Karyadi D. (2016). REVEL: an ensemble method for predicting the pathogenicity of rare missense variants. Am. J. Hum. Genet..

[113] Narasimhan V., Danecek P., Scally A., Xue Y., Tyler-Smith C., Durbin R. (2016). BCFtools/RoH: a hidden Markov model approach for detecting autozygosity from next-generation sequencing data. Bioinformatics.

[114] Zhan X., Hu Y., Li B., Abecasis G.R., Liu D.J. (2016). RVTESTS: an efficient and comprehensive tool for rare variant association analysis using sequence data. Bioinformatics.

[115] Kamat M.A., Blackshaw J.A., Young R., Surendran P., Burgess S., Danesh J., Butterworth A.S., Staley J.R. (2019). PhenoScanner V2: an expanded tool for searching human genotype-phenotype associations. Bioinformatics.

[116] McKenna A., Hanna M., Banks E., Sivachenko A., Cibulskis K., Kernytsky A., Garimella K., Altshuler D., Gabriel S., Daly M., DePristo M.A. (2010). The Genome Analysis Toolkit: a MapReduce framework for analyzing next-generation DNA sequencing data. Genome Res..

[117] Whiffin N., Karczewski K.J., Zhang X., Chothani S., Smith M.J., Evans D.G., Roberts A.M., Quaife N.M., Schafer S., Rackham O. (2020). Characterising the loss-of-function impact of 5’ untranslated region variants in 15, 708 individuals. Nat. Commun..

[118] Wright S. (1922). Coefficients of inbreeding and relationship. Am. Nat..

[119] Durinck S., Spellman P.T., Birney E., Huber W. (2009). Mapping identifiers for the integration of genomic datasets with the R/Bioconductor package biomaRt. Nat. Protoc..

[120] Yin X., Chan L.S., Bose D., Jackson A.U., VandeHaar P., Locke A.E., Fuchsberger C., Stringham H.M., Yu K., Silva L.F. (2021). Genome-wide association study of 1,391 plasma metabolites in 6,136 Finnish men identifies 303 novel signals and provides biological insights into human diseases 10. Preprint at medRxiv.

[121] Surendran P., Stewart I.D., Au Yeung V.P.W., Pietzner P., Raffler J., Wörheide M.A. (2022). Rare and common genetic determinants of metabolic individuality and their effects on human health. Nat Med..

[122] Do K.T., Wahl S., Raffler J., Molnos S., Laimighofer M., Adamski J., Suhre K., Strauch K., Peters A., Gieger C. (2018). Characterization of missing values in untargeted MS-based metabolomics data and evaluation of missing data handling strategies. Metabolomics.

[123] Cao Z., Wang L., Chen Y., Cai R., Lu J., Yu Y., Chen C., Gu F., Yang J., Ma X. (2017). VarfromPDB: an automated and integrated tool to mine disease-gene-variant relations from the public databases and literature. J. Proteonomics Bioinf..

[124] Landrum M.J., Lee J.M., Riley G.R., Jang W., Rubinstein W.S., Church D.M., Maglott D.R. (2014). ClinVar: public archive of relationships among sequence variation and human phenotype. Nucleic Acids Res..

