# Supplementary material for: Identification of PCSK9-like human gene knockouts using metabolomics, proteomics, and whole-genome sequencing in a consanguineous population
Source: Cell Genom. 2022 Nov 15;3(1):100218. doi: 10.1016/j.xgen.2022.100218 (PMC9903797; doi:10.1016/j.xgen.2022.100218)
Supplement: Document S1. Figures S1–S4 and Data S1 [file mmc1.pdf]

Cell Genomics, Volume *3*

Supplemental information

Identification of PCSK9-like human gene knockouts using metabolomics, proteomics, and whole-genome sequencing in a consanguineous population

Aziz Belkadi, Gaurav Thareja, Fatemeh Abbaszadeh, Ramin Badii, Eric Fauman, Omar M.E. Albagha, The Qatar Genome Program Research Consortium, and Karsten Suhre

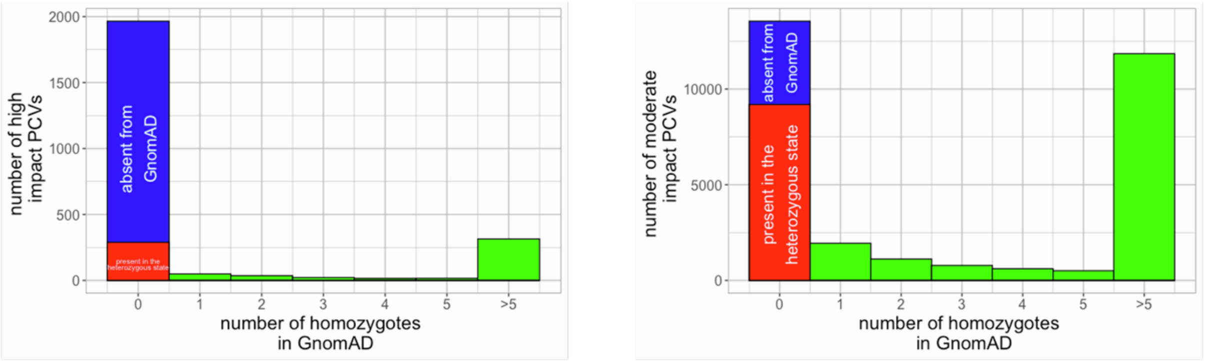

Figure S1. Distribution of high impact homozygous PCVs (left) and moderate impact homozygous PCVs (right), Related to Figure 1. Over 125,748 participants of the GnomAD project. Nearly all the high impact PCVs were not detected in the GnomAD prject. The PCVs completely absent from GnomAD are shown in blue, those present in GnomAD exclusively in the heterozygous state are shown in red, and those identified in the homozygous state in GnomAD are shown in green.

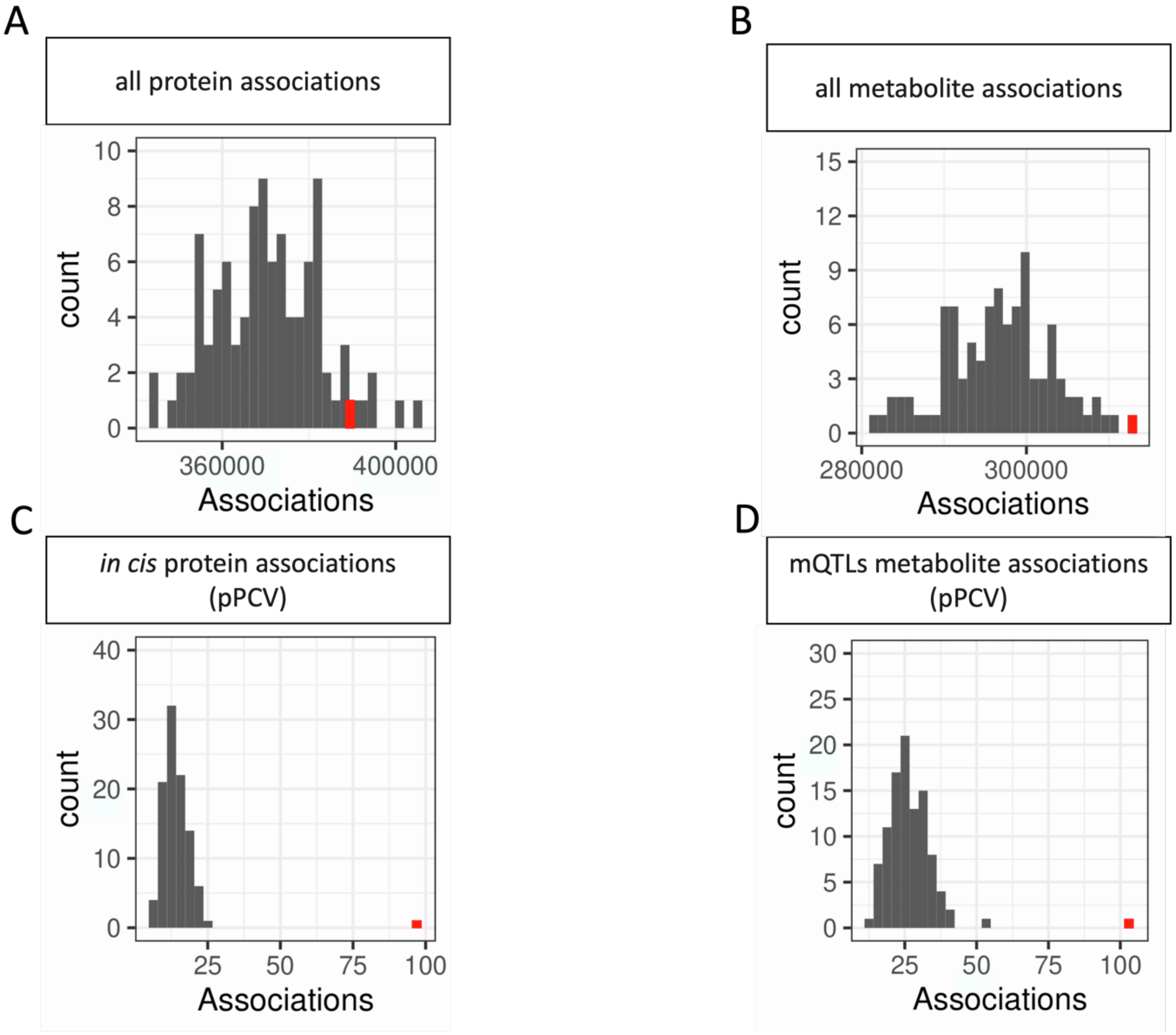

Figure S2. False discovery rate (FDR) for, Related to Table S2, Table S3, Table S4, Table S5 and Methods. A) all PCV/protein associations, B) all PCV/metabolite associations, C) *in cis* protein associations: PCVs affecting the proteins determined (pPCVs), and D) metabolite associations reported in mGWAS (mPCVs). The red bar indicates the number of associations. The gray bars show the numbers of associations by sampling. The FDR was estimated by dividing the mean number of associations obtained by sampling by the total number of associations.

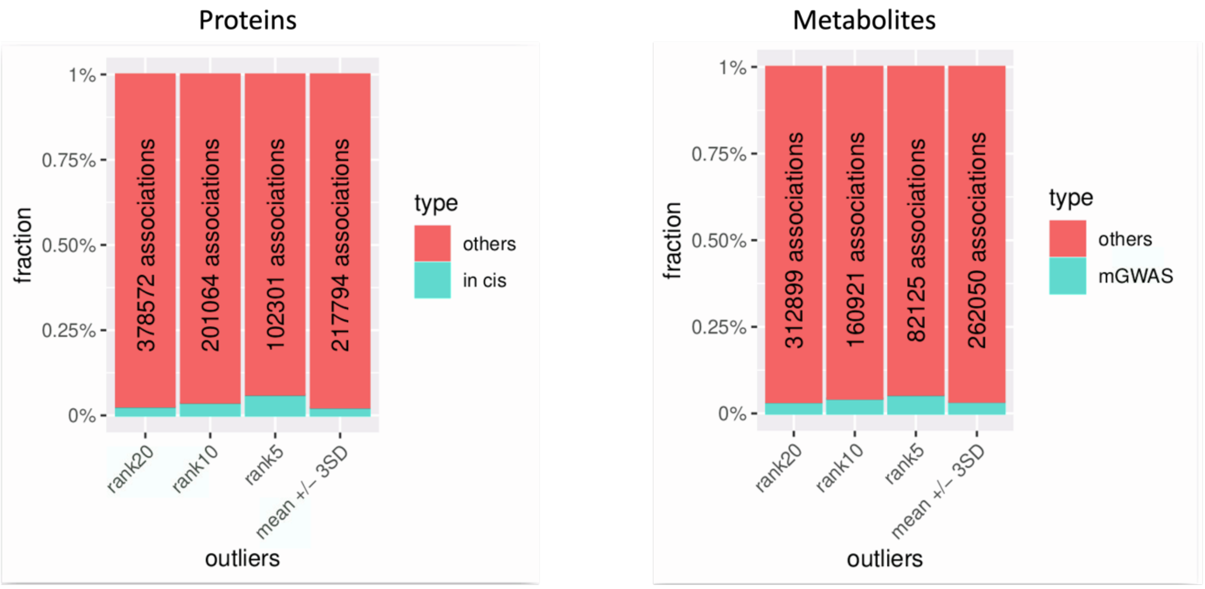

Figure S3. Effect of extreme threshold on pPCVs and mPCVs, Related to Figure 3 and Methods. In both proteins and metabolites, 4 extreme thresholds were compared: the 20 extreme, the 10 extreme, the 5 extreme and the mean ± 3 standard deviations. Represented here the 1% of the associations identified with every threshold. pPCVs (in cis associations, on the left) and mPCVs (metabolite associations reported in the mGWAS, on the right) are colored in green and the rest of the associations is colored in red.

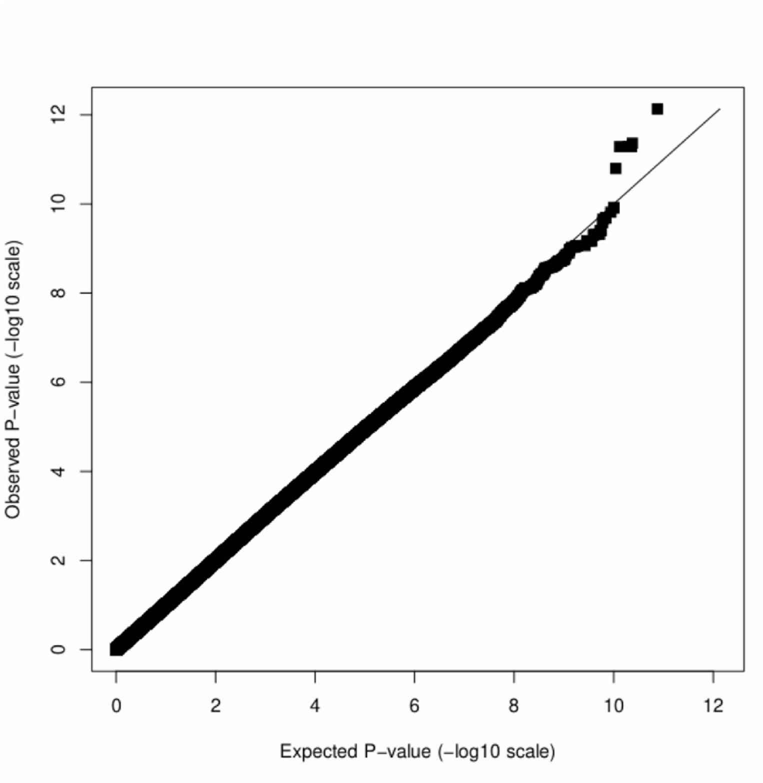

Figure S4. Quantile-Quantile plot of the extreme protein and metabolite associations, Related to Table S2, Table S3 and Methods. In this plot, each dot corresponds to a protein changing variant, carried by at least two homozygotes and up to five homozygotes in QBB, tested for association with phenotypes: proteins metabolites and laboratory data. The x-axis represents the expected -log10 p values under the null hypothesis and the y-axis represents the observed - log10 p values. Upper right dots with higher observed significance than expected represent candidate variants for association with the phenotype tested.

Data S1

We describe here, in greater detail, the showcases highlighted in the paper (Table 1). For each case, we present a vignette beginning with a high-level summary (in bold type-face), followed by a detailed description of all the available evidence and related information and a figure showing key data plots. All the protein, metabolite and clinical biochemistry data are presented on plots with a normalized scale (Z-score, mean = 0, SD = 1). For 10 cases for which more than one homozygote carrier was present, we identified additional significant associations. For this purpose, we applied an approximate P-value as described in the Methods section.

Vignette 1: PCSK9 – LDL-cholesterol

We identified two PCSK9 missense variants associated with low PCSK9 protein levels and low LDL-cholesterol levels circulating in the blood. The first variant was studied in detail and found to be associated with a lowering of LDL-C level and a 50% decrease in the incidence of coronary heart disease. The second variant, reported here for the first time in the homozygous state, was carried in the heterozygous state by one European from the UKB and one African from the GnomAD. This second PCSK9 variant has similar PCSK9- and LDL-C level-lowering effects to the first variant.

The proprotein convertase subtilisin/kexin type 9 (PCSK9) protein plays a key role in cholesterol homeostasis by directing membrane-bound low-density lipoprotein cholesterol (LDL-C) receptors for degradation in the lysosome. In the QBB, we identified one homozygote and 30 heterozygotes for rs11591147 and one homozygote and eight heterozygotes for rs746442570. PCSK9 level ranked 5th of 2,935 variants for the rs11591147 homozygote and 6th of 2,935 for the rs746442570 homozygote (P = 1.39e-05) (Figure 4B). The effect of the variants appears to be additive, with the overall PCSK9 levels for heterozygotes of both variants being lower than the wild-type values and higher than those of homozygotes (Figure 4D). The PCSK9 variants had a strong effect on LDL-C metabolism: both PCSK9 homozygotes had very low LDL-C levels in the blood (P = 1.26e-04) (Figure 4A), whereas heterozygotes had LDL-C levels intermediate between the homozygous and wild-type levels (Figure 4C).

The missense variant rs11591147 has attracted considerable attention from the scientific community interested in genetic variants causing PCSK9-knockout and lowering LDL-C levels. This attention reflects the promising discoveries made for this variant, which has been

significantly associated with a decrease in plasma LDL-C levels and a decrease in the risk of incident cardiovascular disease [43,67]. This variant is more frequent in European than in other populations [16,43,68–70]. For instance, rs11591147 was recently shown to be associated with plasma LDL-C levels in Europeans, but not in East Asians, due to its lower frequency in non-European populations [71]. Furthermore, 86 homozygotes for rs11591147 were identified in the UKB and 35 of the 37 homozygotes for rs11591147 identified in GnomAD were European, whereas no homozygotes for rs746442570 were identified in either of these datasets. PCSK9 provides proof-of-concept of our strategy for identifying PCVs causing extreme blood phenotypes.

Vignette 2: Four genes affecting betaine metabolism

We identified one homozygote for a variant of BHMT (the enzyme responsible for betaine degradation) and two homozygotes for a variant of SLC6A12 (the betaine transporter), all with high levels of betaine. Two other QBB participants, homozygous for variants of CBS and SLC6A5, also had high betaine levels but high levels of dimethylglycine (the product of betaine degradation), contrasting with the carriers of the other two variants. As betaine and vitamins are the treatment of choice for CBS deficiency, we suspect that high betaine levels are associated with either betaine intake or a healthy diet of the participant.

Betaine homocysteine S-methyltransferase (BHMT) is a zinc-metalloenzyme responsible for the transfer of a methyl group from trimethylglycine (betaine) to produce dimethylglycine, and a hydrogen ion from homocysteine to generate methionine (Figure vignette 2A). We identified one homozygote for the missense variant at chr5:78417119 with a high betaine level (Figure vignette 2B) in QBB. Two other QBB participants were found to carry a homozygous in-frame deletion within the sodium- and chloride-dependent betaine transporter gene (SLC6A12) at chr12:301795. Betaine levels were high in the two SLC6A12 homozygotes (Figure vignette 2B). The QBB participant with the highest betaine level carried a homozygous missense variant (rs398123151) of the cystathionine beta synthase (CBS). CBS is the enzyme responsible for the degradation of homocysteine to cystathionine (Figure vignette 2A). We identified one other QBB participant carrying a missense variant (rs543307278) of SLC6A5. This participant also had high betaine levels (Figure vignette 2B).

None of the homozygotes for BHMT and SLC6A12 had extreme homocysteine levels, probably due to the existence of other homocysteine degradation pathways. Data concerning

homocysteine levels were missing for the CBS and SLC6A5 homozygotes, possibly by chance (Methods, Fisher $P = 0.28$ and 0.02 for CBS and SLC6A5 homozygotes, respectively). Both BHMT and SLC6A12 homozygotes had low dimethylglycine levels, whereas the CBS and the SLC6A5 homozygotes had high dimethylglycine levels (Figure 2C). Only the CBS and SLC6A5 homozygotes had high methionine levels (Figure vignette 2D).

We assessed the betaine levels of individuals heterozygous for the BHMT, SLC6A12, CBS and SLC6A5 variants. Heterozygotes for the BHMT and SLC6A12 variants had higher betaine levels than wild-type individuals. Heterozygotes for the CBS variant had lower betaine levels than wild-type individuals. No difference was observed between wild-type individuals and individuals heterozygous for the SLC6A5 variant (Figure vignette 2E).

Betaine, dimethylglycine and methionine levels were similar in individuals homozygous for the CBS and SLC6A5 variants. Dimethylglycine and methionine levels in CBS and SLC6A5 homozygotes differed significantly from those in individuals homozygous for the BHMT and SLC6A12 variants. Furthermore, neither heterozygotes for the CBS variant nor heterozygotes for the SLC6A5 variant had higher betaine levels than wild-type individuals. These observations suggest that different pathways underlie the high betaine levels in homozygotes for the CBS variant and homozygotes for the SLC6A5 variant. For instance, there are no homozygotes for the CBS variant identified here (rs398123151) in either UKB or GnomAD. In various studies, rs398123151 has been shown to be associated with homocystinuria (OMIM #236200), a familial metabolic disorder caused by methionine synthase deficiency [72–75]. Patients with this condition are treated with a low-methionine diet, vitamin B6, folate, vitamin B12, and, most importantly, betaine. Betaine is administered for homocystinuria treatment, to decrease homocysteine levels by promoting the conversion of homocysteine back into

methionine by BHMT. Unlike the homozygotes for the BHMT and SLC6A12 variants, the homozygotes for the CBS and SLC6A5 variants had high dimethylglycine and methionine levels, suggesting strong betaine degradation via the BHMT pathway in these participants. The individual homozygous for the CBS variant is a 21-year-old man who takes vitamins B and D and eats a vegan diet due to an undefined disease. We hypothesized that the high levels of betaine in the homozygote for the CBS variant were caused by the diet of this participant and/or unreported betaine intake to treat the homocystinuria caused by CBS deficiency.

The SLC6A5 missense variant (rs543307278) was carried by three South Asian homozygotes in GnomAD and was not present in the homozygous state in the UKB. We found no link between homozygosity for the SLC6A5 variant and high betaine levels in one 47-year-old man without a specific diet. However, the similar patterns observed for the CBS and SLC6A5 homozygotes suggested that the high betaine levels in these individuals were caused by betaine intake. Further investigations are required to confirm our hypotheses and to assess the role of SLC6A5 in betaine degradation.

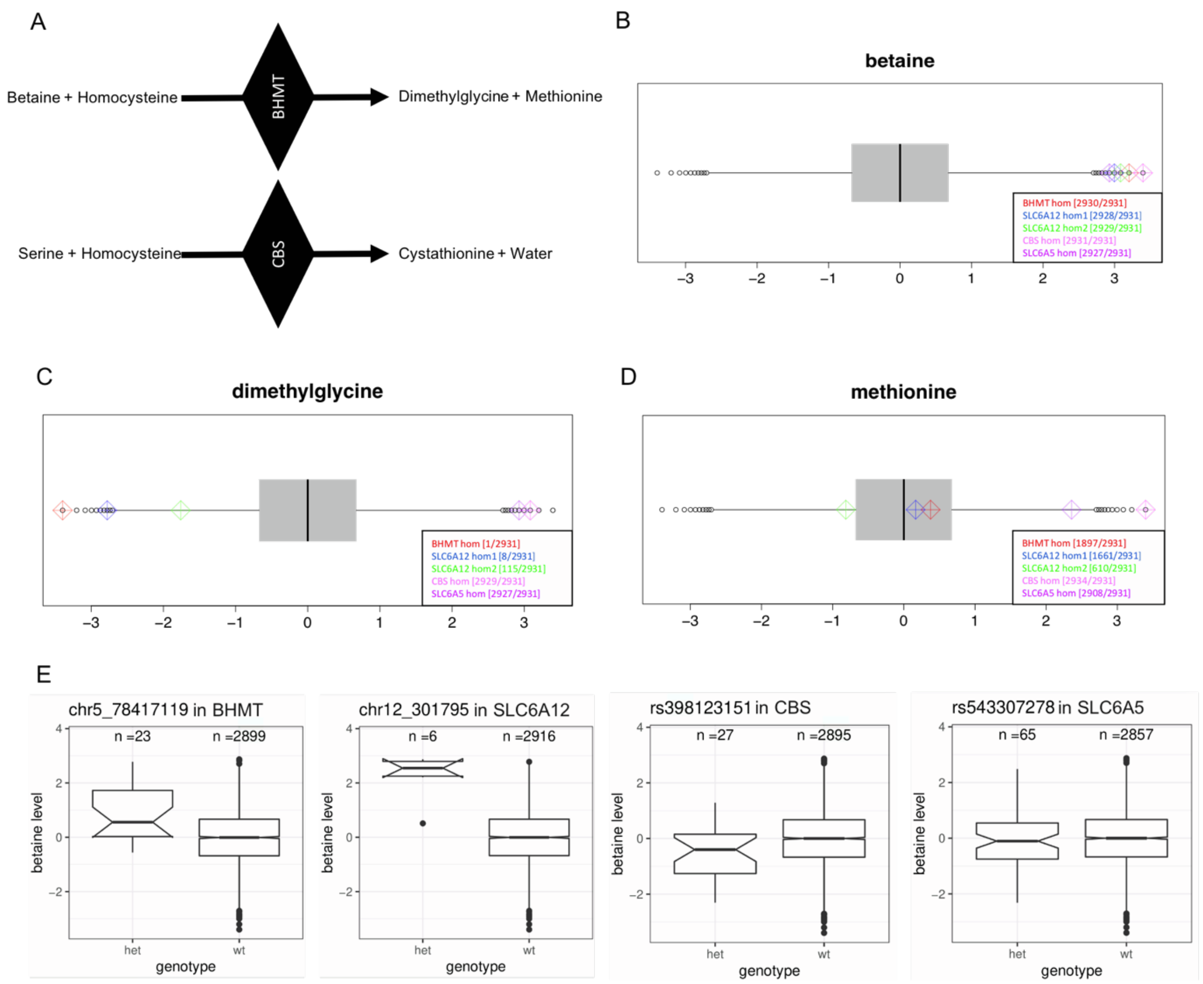

Figure vignette 2. High betaine levels in carriers of the BHMT, SLC6A12, CBS and SLC6A5 variants. A) Pathway of homocysteine remethylation by BHMT and the trans-sulfuration of homocysteine by CBS. B) The betaine levels in one BHMT, two SLC6A12, one CBS and one SLC6A5 homozygote were the highest in the QBB. C. Dimethylglycine levels were low in BHMT and SLC6A12 homozygotes and high in CBS and SLC6A5 homozygotes. C) Methionine levels were high in CBS and SLC6A5 homozygotes only. D) Betaine levels in BHMT, SLC6A12, CBS and SLC6A5 heterozygotes and wild-type individuals. Overall, betaine levels were higher in BHMT and SLC6A12 heterozygotes than in wild-type individuals.

Vignette 3: ACY1 – acetylated amino acids

We identified two homozygotes for ACY1 variants with low ACY1 levels and high acetylated amino-acid levels but normal free amino-acid levels. The first variant has already been reported to be associated with ACY1 deficiency, whereas the second variant is new. Another QBB participant not homozygous for an ACY1 variant had high levels of both acetylated and free amino acids, probably due to a protein-rich diet.

Aminoacylase-1 (ACY1) catalyzes the hydrolysis of acylated N-amino acids and acts preferentially on aliphatic acetylated amino acids, such as methionine, in particular. For ACY1, we identified one homozygote for rs121912698 and another homozygote for rs2229152. These two individuals homozygous for ACY1 variants had the lowest ACY1 levels in the QBB (P = 9.29e-7) (Figure 5A). Heterozygotes for these two missense variants had lower ACY1 levels than wild-type individuals (Figure 5B).

The levels of acetylated aliphatic amino acids, such as acetylmethionine, acetylalanine, acetylisoleucine, acetylleucine and acetylvaline were high in individuals homozygous for ACY1 variants (P < 2.29e-05) (Figure 5C and Table S7). The levels of acetylglutamate, acetylhistidine, acetylserine and acetylthreonine were also high in the two ACY1-homozygotes (P < 5.92e-04). The levels of the other acetylated amino acids — acetylglycine, acetylproline, acetylaspartate, acetyltyrosine, acetylasparagine, acetyltryptophan, acetyllysine, acetylarginine, acetylphenylalanine and acetylhistamine — were not extreme in ACY1- variant homozygotes (P > 0.001) (Table S7).

We identified another QBB participant with high acetylated amino-acid levels (Figure 5C and Table S7). This participant was not homozygous for any identified ACY1 PCV. We therefore

investigated possible compound heterozygosity in this individual. We found only one heterozygous ACY1 missense variant (rs34017492) in this participant.

The rs34017492 heterozygote had high levels of all the acetylated amino acids present at high levels in the two ACY1 homozygotes except for acetylglycine. However, this participant also had high levels of acetyltyrosine, acetyltryptophan, acetyllysine, acetylarginine and acetylphenylalanine (Table S7).

We investigated the free amino-acid levels of the two ACY1 homozygotes and the rs34017492 heterozygote (Figure 5D and Table S7). Neither of the ACY1 homozygotes had extreme free amino-acid levels. By contrast, 13 of the 18 free amino-acid levels measured in the QBB were high in the rs34017492 heterozygote. The rs34017492 heterozygote reported following a strict low-fat, high-protein diet to lose weight. Unlike those in the ACY1 homozygotes, the high acetylated and free amino-acid levels in the rs34017492 heterozygote may, therefore, be due to diet rather than genetic consequences.

ACY1 deficiency (OMIM #609924) causes a metabolic disorder due to acetylated amino-acid accumulation in the brain. ACY1 deficiency has incomplete penetrance and the pattern and severity of symptoms vary considerably between affected individuals. ACY1-deficient individuals have mutations of the ACY1 gene and high levels of acetylated amino acids, but some nevertheless display normal, healthy development. Other patients suffer from recurrent seizures and severely delayed psychomotor development. Seven variants causing ACY1 deficiency have been described to date [23–26]. One of the two missense variants identified here, rs121912698, is a well-established variant causing ACY1 deficiency. The rs121912698 variant is carried by four homozygotes in GnomAD. We identified a second missense variant in this

study, rs2229152, which is carried by three homozygotes in GnomAD and has not been associated with ACY1 deficiency. This variant was associated with low levels of ACY1 and high levels of acetylated amino acids in the blood. The rs2229152 variant identified here should therefore potentially be included in the list of mutations for ACY1 deficiency screening.

ACY1 is also associated with type 2 diabetes (T2D). Three different protein association studies have reported that high levels of ACY1 are associated with a low risk of T2D [28–30]. Ngo et al. found that ACY1 levels are, indeed, inversely associated with N-acetyl amino-acid substrates and positively associated with free amino-acid products in human plasma [30]. We confirmed the correlation between ACY1 levels and the ratio of methionine to acetylmethionine levels (Figure 5E). However, studies of ACY1 overexpression in mouse models have consistently demonstrated ACY1 levels to be inversely associated with insulin resistance and blood glucose levels [30].

Vignette 4: Plasmin and excessive blood coagulation

PLG is a gene that encodes three proteins: 1) plasminogen (plasmin zymogen), 2) plasmin (the active enzyme), and 3) angiostatin (the plasmin cleavage product). The levels of these three proteins in QBB participants were determined with the Somascan kit. We identified one individual homozygous for a PLG variant with high levels of plasminogen and angiostatin, and normal levels of plasmin. According to the medication questionnaire, the individual homozygous for the PLG variant was on warfarin treatment, probably to treat hypercoagulability or a related disease. Warfarin decreases the levels of four coagulation factors. The levels of these four coagulation factors were low in the individual homozygous for the PLG variant. In addition, this homozygote had a prolonged partial thromboplastin time (APTT) and prothrombin time (PT) and a high international normalized ratio (INR), probably due to the warfarin treatment.

Plasmin (encoded by the PLG gene) is an important enzyme involved in the degradation of fibrin clots. Plasminogen is an essential precursor of plasmin that must bind to clots, or to the cell surface to be converted into active plasmin (Figure 6A). This activated plasmin promotes angiogenesis. The conversion of plasminogen into plasmin involves cleavage of the peptide bond between Arg-561 and Val-562. In the presence of free sulfhydryl donors, plasmin is converted into the angiogenesis inhibitor angiostatin (Figure 6A). We identified one PLG missense variant, rs4252129, carried by one homozygote in the QBB. Both plasminogen and angiostatin levels were low in this participant who, surprisingly, had normal plasmin levels (Figure 6B). The heterozygotes for this missense variant included in the QBB had lower plasminogen and angiostatin levels than the wild-type individuals. Plasmin levels were similar in rs4252129 heterozygotes and wild-type individuals (Figure 6C).

The QBB participants were asked to complete self-reported medication questionnaires. The rs4252129 homozygote, a 53 year-old man, reported warfarin treatment on this questionnaire. This individual had a prolonged partial thromboplastin time (APTT) and prothrombin time (PT) and a high international normalized ratio (INR) (Figure 6D), probably related to warfarin treatment. Furthermore, the rs4252129 homozygote had low levels of four coagulation factors (Figure 6E): coagulation factor VII (F7), thrombin (F2), coagulation factor IX (F9) and coagulation factor X (F10). Two other coagulation factors — coagulation factor V (F5) and coagulation factor XI (F11) — were present in the rs4252129 homozygote at levels within the normal range. Warfarin is used to treat thromboses. It acts by inhibiting the vitamin K cycle by targeting the vitamin K oxide reductase enzyme (VKOR) [76]. In the absence of sufficient active vitamin K, coagulation factors F2, F7, F9, and F10 are less able to clot the blood. Like the results of blood coagulation tests, the low levels of these coagulation factors in this homozygote could be explained by warfarin intake.

Sixteen homozygotes for rs4252129 were reported in GnomAD. *In vitro* analysis [77] showed that the plasminogen activation site mutant p.Arg561Ala was not cleaved by plasminogen activators, preventing the conversion of plasminogen into plasmin. Another plasminogen mutant affecting in the functional serine protease domain p.Asp646Glu is cleaved by plasminogen activators, but the resulting two-chain plasmin is inactive due to the substitution of a catalytically essential aspartic acid residue in the serine protease catalytic triad. Angiostatin is not generated in the presence of the plasminogen mutant p.Asp646Glu. The variant identified here, rs4252129 (p.Arg523Trp), affects the fifth Kringle domain, a region excised during plasmin-to-angiostatin conversion (Figure 6A).

We have no direct information about the medical conditions presented by the rs4252129 homozygote, but his treatment with warfarin suggests that he suffers from a of blood-clotting problem. Both homozygous and heterozygous carriers of rs4252129 have normal plasmin levels and low plasminogen and angiostatin levels. Our findings suggest there may be unknown regulatory mechanisms controlling plasmin levels in the blood.

<u>Vignette 5:</u> ACSM2A and indolepropionic acid

We identified six homozygotes for ACSM2A PCVs: four homozygotes for a stop-gain variant (rs59261767), one homozygote for a missense variant (chr16:20480888) and one homozygote for another missense variant (rs369633543). The six individuals homozygous for ACSM2A variants had high levels of indolepropionic acid (IPA) and phenylpropanoic acid (PPA). **ACSM2A** ***encodes a mitochondrial enzyme involved in fatty-acid metabolism. The link between ACSM2A and the two metabolites remains unknown. However, both metabolites are synthesized by the gut microbiota, suggesting that ACSM2A may affect bacterial function. IPA has been studied for its protective role against many diseases including Alzheimer's disease, type 2 diabetes and non-alcoholic fatty liver disease.***

Acyl-CoA synthetase medium-chain family member 2a (ACSM2A) encodes an acyl-coenzyme A synthetase that catalyzes fatty-acid activation. We identified one ACSM2A stop-gain variant carried by four homozygotes (rs59261767), one ACSM2A missense variant carried by one homozygote (chr16:20480888) and another ACSM2A missense variant carried by one homozygote (rs369633543). Indolepropionic acid (IPA) levels were high in individuals homozygous for ACSM2A variants (P = 4.92e-15. Figure vignette 5A). The detection of IPA in human blood results from tryptophan degradation by various gut bacteria [78,79]. However, in complex bacterial communities, IPA levels are dependent on *C. sporogenes* alone [78]. Overall IPA levels were higher in heterozygotes than in wild-type individuals for the three ACSM2A variants identified (Figure vignette 5C). Five of the six ACSM2A homozygotes also had high levels of phenylpropanoic acid (PPA, P = 1.36e-10. Figure vignette 5B). PPA is generated by the metabolism of phenylalanine by gut bacteria, including *C. sporogenes* [78,79]. Overall PPA levels were higher in heterozygotes than in wild-type individuals for the three ACSM2A variants (Figure vignette 5D).

The individuals homozygous for ACSM2A variants in the QBB had high levels of IPA and PPA. The biological function of PPA remains unknown. IPA provides primary neurons and neuroblastoma cells with full protection against the oxidative damage and death caused by exposure to amyloid B-protein, the inhibition of superoxide dismutase, or treatment with hydrogen peroxide. Amyloid B-protein accumulation is one of the most prominent neuropathologic features of Alzheimer's disease [80]. Furthermore, IPA has a greater hydroxyl radical scavenging capacity than melatonin, an indoleamine considered to be the most potent naturally occurring scavenger of free radicals. IPA has, therefore, been studied for possible therapeutic use in Alzheimer's disease [81–83]. IPA has also been shown to be a pregnane X receptor (PXR) agonist and to play a role in maintaining intestinal barrier function and mucosal homeostasis [84]. Furthermore, IPA has been shown to improve non-alcoholic steatohepatitis in mice, a subtype that can progress to life-threatening conditions, such as cirrhosis and hepatocellular carcinoma [85]. Metabolome-wide association studies with diabetes found a correlation between high levels of IPA in human blood plasma and a lower risk of type 2 diabetes [86,87].

The associations of ACSM2A variants with IPA/PPA levels in six QBB participants and for three different functional variants suggest that ACSM2A silencing is a potential treatment target for increasing IPA/PPA levels in the blood. The link between ACSM2A and high IPA/PPA levels requires further investigation. However, the high levels of these two metabolites synthesized by gut bacteria, including *C. sporogenes,* in individuals homozygous for ACSM2A variants suggest that ACSM2A may act on the function of the bacteria.

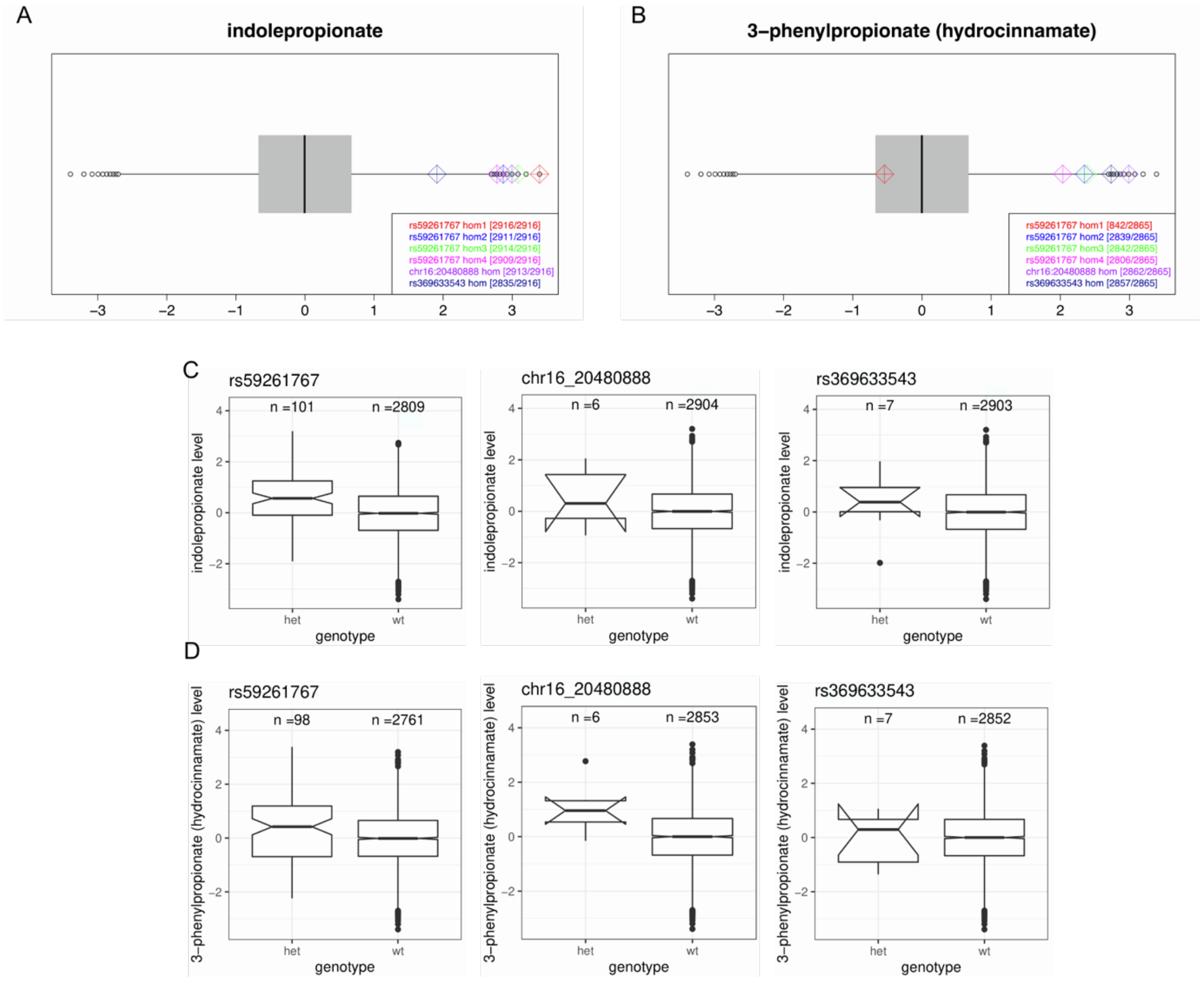

Figure vignette 5. Carriers of ACSM2A stop-gain (rs59261767), and missense (chr16:20480888 and rs369633543) variants have high levels of indolepropionic acid (IPA) and phenylpropanoic acid (PPA). A) Six individuals homozygous for ACSM2A variants have high levels of IPA. B) Five individuals homozygous for ACSM2A variants have high levels of PPA. C) The heterozygotes for ACSM2A variants in the QBB have higher levels of IPA than wild-type individuals. D) The heterozygotes for ACSM2A variants in the QBB have higher levels of PPA than wild-type individuals.

Vignette 6: ABCG5 and plant sterols

We identified three homozygotes for ABCG5 variants: two homozygotes for one missense variant (rs145164937) and one homozygote for another missense variant (rs569748582). These three individuals homozygous for ABCG5 variants had high levels of two plant sterols: campesterol and sitosterol. ABCG5 and its paralog, ABCG8, are responsible for the efflex of sterols, including cholesterol, from enterocytes and hepatocytes into the intestine and bile, respectively. ABCG5/ABCG8 loss-of-function variants cause sitosterolemia, whereas gain-of-function variants confer a higher risk of cholesterol gallstones. Studies in animal models have shown that ABCG5/ABCG8 loss-of-function decreases biliary cholesterol levels. Further validations of these observations in humans are required.

ATP binding cassette subfamily G member 5 (ABCG5) and its paralog ABCG8 are sterol efflux transporters that play a key role in the hepatic secretion and intestinal absorption of cholesterol and plant sterols. We identified one ABCG5 missense variant (rs145164937) carried by two homozygotes and another ABCG5 missense variant (rs569748582) carried by one homozygote. The levels of two plant sterols, including campesterol (P = 2.48e-07) and sitosterol (P = 9.40e-07) were high in all three ABCG5 variant homozygotes (Figure vignette 6A and 6B). Individuals heterozygous for rs145164937 had higher levels of campesterol and sitosterol than wild-type individuals (Figure vignette 6C and 6D). No heterozygote for rs569748582 was identified in the QBB.

Loss-of-function mutations of *ABCG5* and *ABCG8* cause sitosterolemia (OMIM #618666), a rare inherited lipid storage disease characterized by a significant increase in the concentrations of plant sterols (sitosterol, campesterol, stigmasterol, and avenosterol) in the blood and tissues

[88]. The clinical outcome of sitosterolemia may include high LDL-C levels, premature coronary artery disease and death, hemolytic anemia, macrothrombocytopenia, splenomegaly, adrenal dysfunction, high liver enzyme levels, and cirrhosis [89]. Both the ABCG5 missense variants identified here are rare, and only one homozygote for rs145164937 was reported in GnomAD. As both variants were associated with high levels of the two plant sterols determined, campesterol and sitosterol, they should be considered an etiology of sitosterolemia.

Cholesterol gallstones are one of the commonest and most costly digestive diseases worldwide [90]. Clinical outcomes are predicted for one third of individuals with gallstones [91]. The missense variant rs11887534 of ABCG8, which may be gain-of-function [92] was associated with markedly low serum levels of the plant sterols campesterol and sitosterol [93,94] and a higher risk of gallstone disease [95,96]. The ABCG5/ABCG8 intronic variant rs6544713 was also recently shown to be associated with lower campesterol levels and a higher risk of gallstones [62]. Yin et al. explained that a decrease in biliary cholesterol levels decreases the risk of gallstones due to competition between campesterol and cholesterol for ABCG5/ABCG8 transporters during biliary cholesterol secretion. ABCG5/ABCG8 knockout in mice increases plasma cholesterol levels, but decreases biliary cholesterol levels [97]. The individuals homozygous for ABCG5 variants in the QBB had normal cholesterol levels. Complementary analyses are required to validate the hypothesis that the two ABCG5 missense variants identified here decrease biliary cholesterol levels and the risk of cholesterol gallstones.

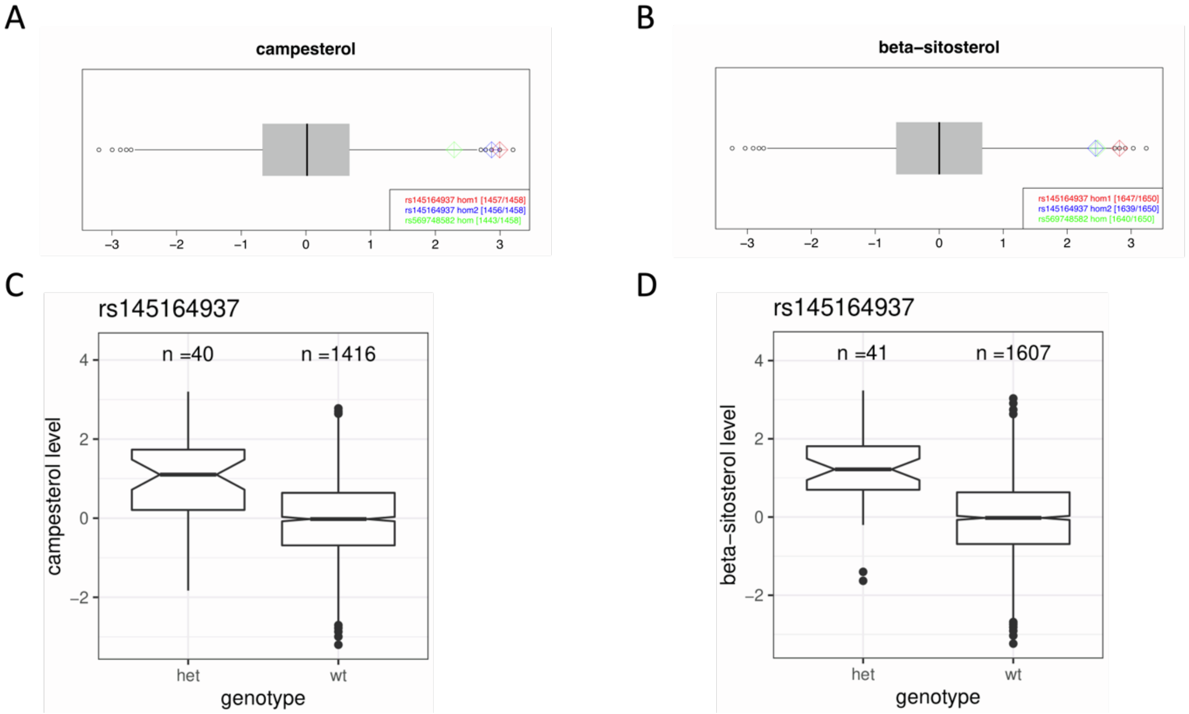

Figure vignette 6. ABCG5 variant carriers have high levels of plant sterols. Two homozygotes for the ABCG5 missense variant rs145164937 and one homozygote for the ABCG5 missense variant rs569748582 have high levels of the plant sterols A) campestrol and B) beta-sitosterol. Heterozygotes for the ABCG5 missense variant rs145164937 in the QBB have higher levels of C) campestrol and D) beta- sitosterol than wild-type individuals.

Vignette 7: ABCC2 and glycolic acid sulfate

We identified four homozygotes for ABCC2 variants. Three had high levels of glycolic acid sulfate and three other unknown metabolites. The fourth homozygotes for an ABCC2 variant had high levels of two unknown metabolites and moderately high levels of another two metabolites potentially explicable by atenolol intake. ABCC2 is involved in the metabolism of certain drugs, through the clearance of xenobiotics from organs. ABCC2 inhibition leads to high intracellular drug concentrations, and genetic variants of ABCC2 should therefore be taken into account when prescribing ABCC2-related drugs.

Multidrug resistance-associated protein 2 (ABCC2) is mostly expressed in hepatocytes and is involved in bile acid transport across the extra- and intra-cellular matrix. ABCC2 deficiency underlies Dubin-Johnson syndrome (OMIM # 237500), a rare, autosomal recessive, benign disorder characterized by high bilirubin levels in the blood.

We identified two ABCC2 missense variants: rs867979691 and rs140680467. Each variant was carried by two homozygotes in the QBB. All homozygotes for ABCC2 variants had high levels of X - 21471 (P = 2.97e-10) and X - 21467 (P = 1.32e-09, Figure vignette 7A). Both rs867979691 homozygotes and one rs140680467 homozygote had high levels of glycolic acid sulfate (P = 3.42e-08) and X - 21441 (P = 2.56e-08, Figure vignette 7A). Both the heterozygotes for ABCC2 variants had higher levels of X - 21441, X - 21467, X - 21471 and glycolic acid sulfate than wild-type individuals (Figure vignette 7B).

Only two homozygotes for rs140680467 were reported in GnomAD, which contained no homozygotes for rs867979691. ABCC2 is a biliary transporter, potentially accounting for its

association with the bile acid glycolic acid sulfate [42]. Associations of ABCC2 variants with three other unknown metabolites, X - 21441, X - 21467 and X – 21471, were reported in a previous mGWAS, suggesting that these unknown metabolites may also be bile acids. Further studies are required to explain the moderately high levels of X - 21441 and glycolic acid sulfate in one rs140680467 homozygote. The use of atenolol to treat hypertension in this patient is one potential explanation, as higher circulating levels of glycolic acid sulfate are associated with the incidence of cardiovascular diseases [98].

ABCC2 is also expressed in the proximal tubule apical membrane in the kidney and has been implicated in drug metabolism through a role in transporting substances out of cells. Various commercially available drugs inhibit ABCC2, potentially resulting in high intracellular levels of other drugs transported by ABCC2 in cases of multiple-drug interactions [99,100]. Hence, genetic variants of ABCC2 for which there is evidence of protein function disruption, like the two variants identified here, should be taken into account when determining the dose of ABCC2-related drugs to be prescribed.

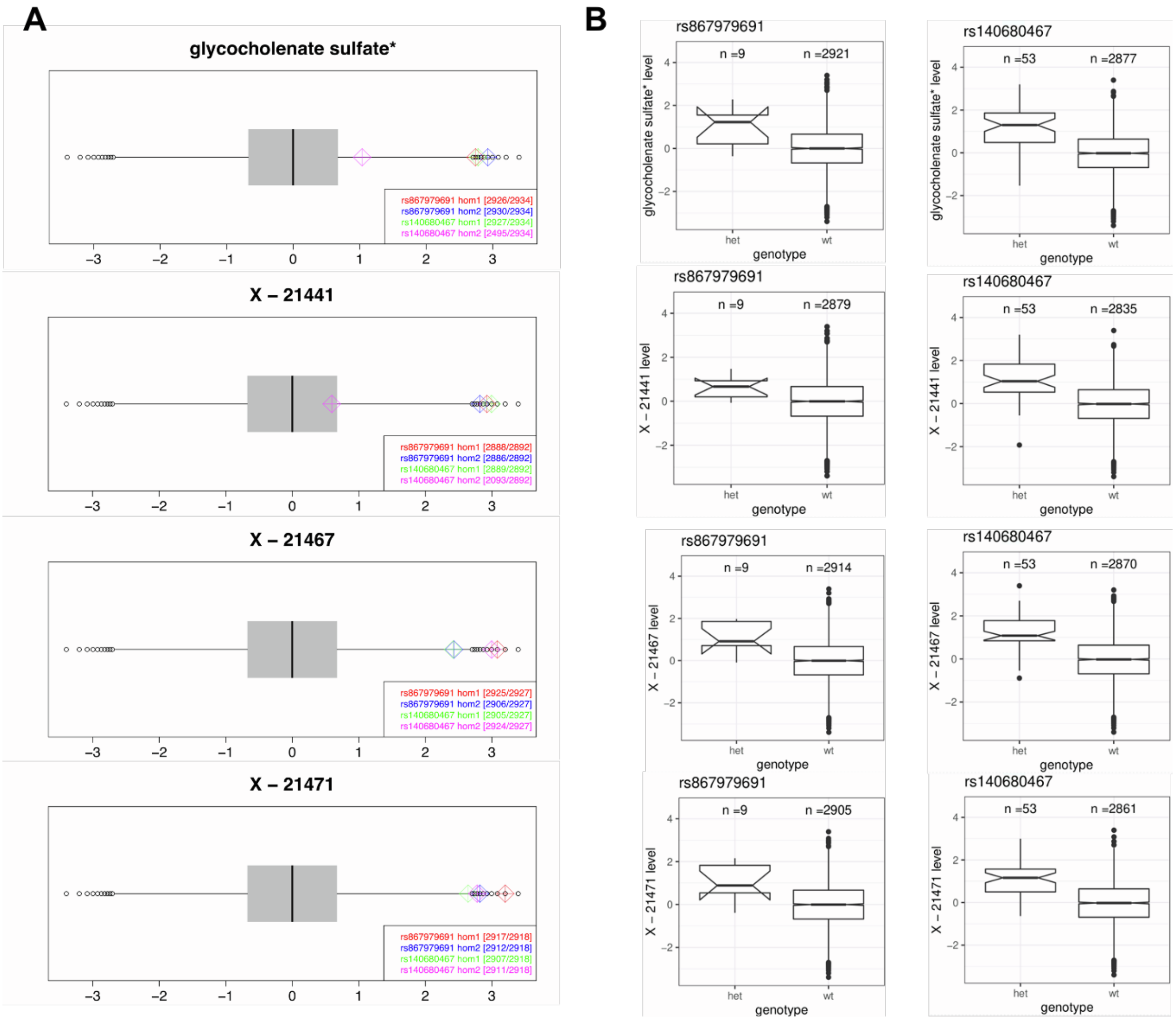

Figure vignette 7. ABCC2 variant carriers have high levels of bile acids. A) Four individuals in the QBB homozygous for the ABCC2 missense variants rs867979691 and rs140680467 have high levels of the bile acid glycolic acid sulfate and three unknown metabolites, X – 21441, X – 21467 and X - 21471. B) The heterozygotes for the two ABCC2 missense variants, rs867979691 and rs140680467, in the QBB have higher levels of glycolic acid sulfate, X – 21441, X – 21467 and X – 21471 than wild-type individuals.

Vignette 8: PAOX and spermidine-related metabolites

We identified two homozygotes for PAOX variants with high levels of acetylspermidine and two related spermidine metabolites: acisoga and isoputreanine. A recent rare variant-metabolite association study reported an association between CALY variants and acisoga. However, the authors suggested that acisoga levels were actually associated with PAOX rather than CALY, given the close physical proximity of the two genes and existing biochemical knowledge. This hypothesis is confirmed by our findings.

The peroxisomal N(1)-acetyl-spermine/spermidine oxidase (PAOX) is involved in polyamine back-conversion [101]. We identified one PAOX missense variant, rs150446594, carried by two homozygotes in the QBB. Acetylspermidine levels were high in both homozygotes for the PAOX variant (P = 2.05e-05, Figure vignette 8A). We found that the levels of two spermidine-related metabolites were high in the homozygotes for the PAOX variant: acisoga (P = 3.38e-05) and isoputreanine (P = 1.63e-05). Acisoga is a catabolic product of spermidine and isoputreanine is the urinary metabolite of spermidine. Heterozygotes for the PAOX variant had higher levels of acetylspermidine, acisoga and isoputreanine than wild-type individuals (Figure vignette 8B).

Two homozygotes for rs150446594 were reported in GnomAD. A recent rare variant-single metabolite analysis identified an association between calcyon neuron-specific vesicular protein (CALY) variants and acisoga levels [35]. However, the authors proposed that the observed association with polyamines might be better be explained by the nearby PAOX gene, given existing biochemical knowledge. Only one CALY missense variant, rs140691452, carried by one homozygote in the QBB was included in the analysis. The rs140691452 homozygote had no extreme spermidine-related metabolite levels. Our findings therefore confirm the effect of

PAOX variants on polyamine back-conversion through the high levels of three different polyamines in two QBB participants.

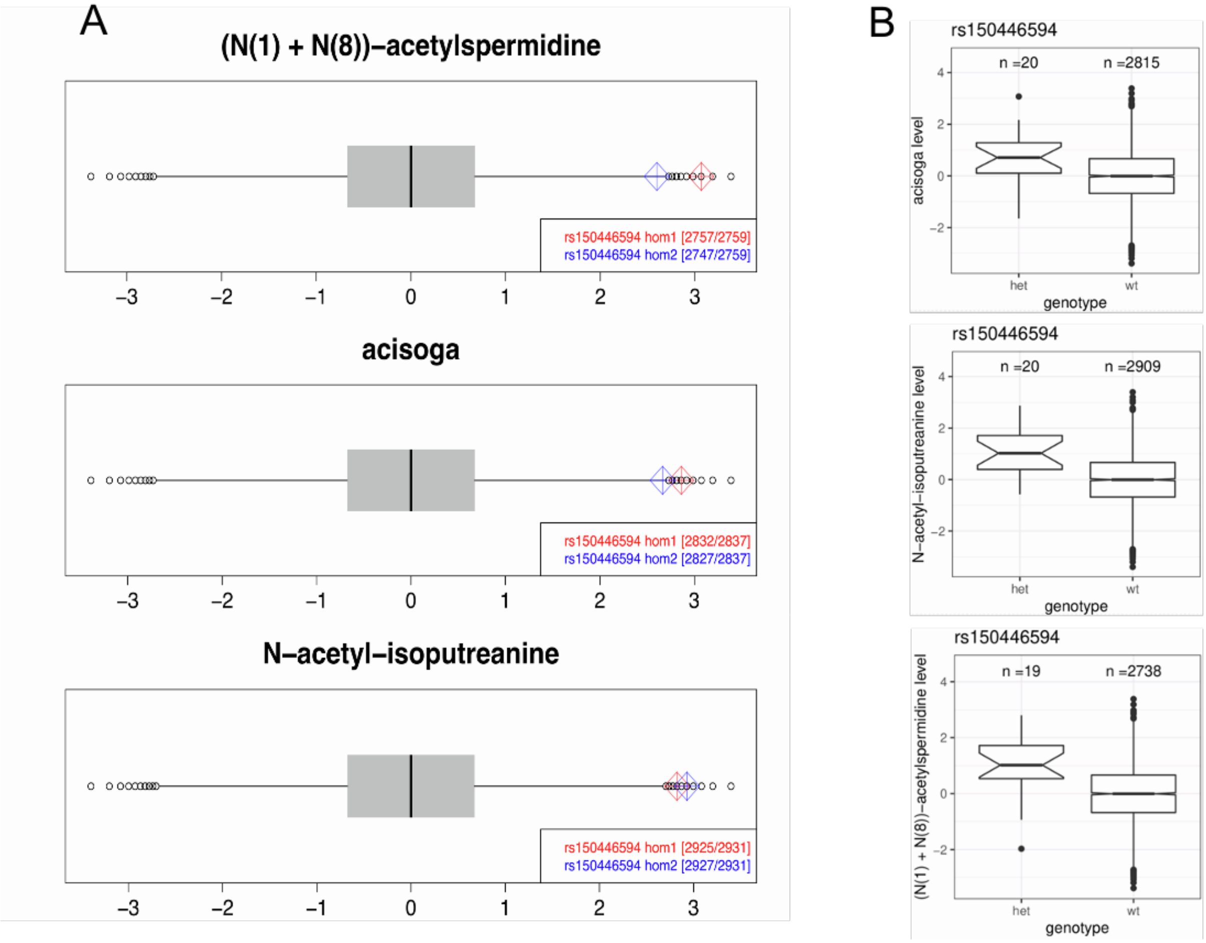

Figure vignette 8. PAOX variant carriers have high levels of spermidine. A) Two individuals homozygous for the PAOX missense variant rs150446594 in the QBB have high levels of three spermidine-related metabolites: acetylspermidine, acisoga and acetylisoputreanine. B) Heterozygotes for the PAOX missense variant rs150446594 have higher levels of acetylspermidine, acisoga and acetylisoputreatine than wild-type individuals.

Vignette 9: AFMID and formylanthranilic acid

We identified four individuals homozygous for an AFMID variant with high levels of formylanthranilic acid. AFMID-knockout mice display impaired glucose tolerance, but we found no association with insulin intolerance in humans homozygous for this AFMID variant.

Arylformamidase (AFMID) is the enzyme responsible for converting formylanthranilic acid into formic acid (Figure vignette 9A). We identified one missense variant of AFMID, rs77585764, carried by four homozygotes in the QBB. All four homozygotes had high levels of formylanthranilic acid (*P* = 9.34e-10, Figure vignette 9B). Heterozygous carriers of rs77585764 had higher formylanthranilic acid levels than wild-type individuals (Figure vignette 9C). AFMID-knockout mice displayed impaired glucose tolerance, despite their insulin sensitivity remaining similar to that in wild-type animals [102]. Further studies of these participants are required to assess the possibility of impaired glucose tolerance due to the AFMID variant identified here.

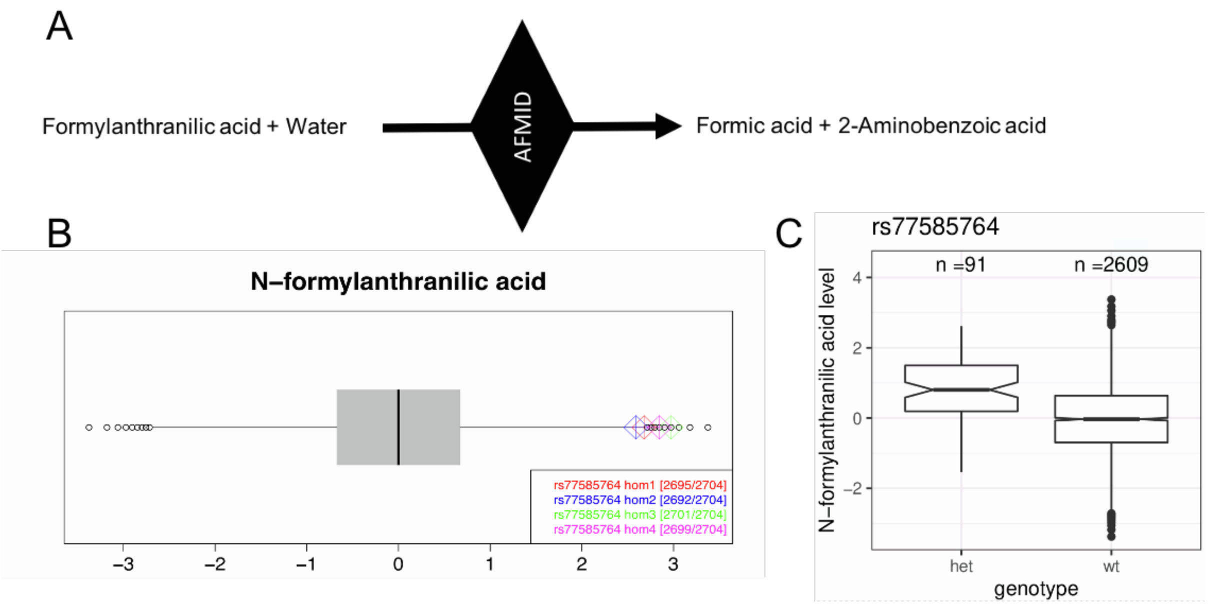

Figure vignette 9. AFMID variant carriers have high levels of formylanthranilic acid. A) AFMID is involved in the hydrolysis of formylanthranilic acid to formic acid in the tryptophan degradation pathway. B) High formylanthranilic acid levels in the four homozygotes for the missense variant rs77585764. C) Heterozygotes for the AFMID missense variant rs77585764 have higher levels of formylanthranilic acid than wild-type individuals.

Vignette 10: UPB1 and ß-ureidopropionate

We identified three homozygotes for UPB1 variants in the QBB, all of whom had high levels of ß-ureidopropionate. Two of these homozygotes had low levels of ß−aminoisobutyrate and ß−aminoisobutyrate was not detected in the third homozygote due to missing homozygosity. Another QBB participant had high ß-ureidoisobutyrate and low ß-aminoisobutanoic acid levels, possibly due to compound heterozygosity for UPB1.

Beta-ureidopropionase (UPB1) is the enzyme that catalyzes the last step in the pyrimidine degradation pathway. UPB1, DHPDH and DHP are the three enzymes responsible for pyrimidine catabolism. The final step of pyrimidine degradation by UPB1 results in the conversion of ß-ureidopropionic acid and ß-ureidoisobutyric acid into ß-alanine and ß-aminoisobutanoic acid, respectively (Figure vignette 10A). We identified one splice acceptor variant, rs138081800, carried by two homozygotes and one missense variant, rs145766755, carried by one homozygote in the QBB. The ß-ureidopropionate levels of these three homozygotes were the highest in the QBB cohort (P = 1.98e-09, Figure vignette 10B). Heterozygotes for both these variants had ß-ureidopropionate levels higher than those of wild-type individuals (Figure vignette 10C). ß-aminoisobutyrate levels were low in two of the three homozygotes (P = 3.73e-06, Figure vignette 10D). No ß-aminoisobutanoic acid determination was available for the remaining homozygote, probably caused by the variant affecting the function of the enzyme in the production of this metabolite (Fisher's test P = 0.0077, see Methods). Such an association was observed only for the variants in the homozygous state (Figure vignette 10E). We identified another QBB participant with high ß-ureidoisobutyrate and low ß-aminoisobutanoic levels (Figure vignette 10B). This participant carried no homozygous PCV for UPB1. We therefore investigated whether he was compound-heterozygous for UPB1. We identified two heterozygous UPB1 missense variants,

rs150338561 and chr22:24919742, in this participant. No other homozygote or compound-heterozygote for these two UPB1 missense variants was identified in the QBB.

UPB1 deficiency (OMIM #606673) is an inborn error of the pyrimidine degradation pathway characterized by a high level of ß-ureidopropionic acid in urine and blood [103]. UPB1-deficient patients have diverse neurological abnormalities, including developmental delay, neurological manifestations (such as ataxia or dystonia), enlarged liver and spleen or gingiva hyperplasia, skeletal dysostosis and prolonged hematological, immunological, ophthalmological and gastrointestinal manifestations. It has been suggested that ß-ureidopropionic acid, one of the accumulating substrates, functions as an endogenous neurotoxin [104]. Both variants identified here have been reported to cause UPB1 deficiency [105,106]. Only one homozygote for rs138081800 was identified in GnomAD. No other homozygotes for rs138081800 or rs145766755 were identified in GnomAD or UKB. The three UPB1 variant homozygotes in the QBB were reported to be in good health and not suffering from any neurological disorders. This suggests an incomplete penetrance for these two UPB1 variants.

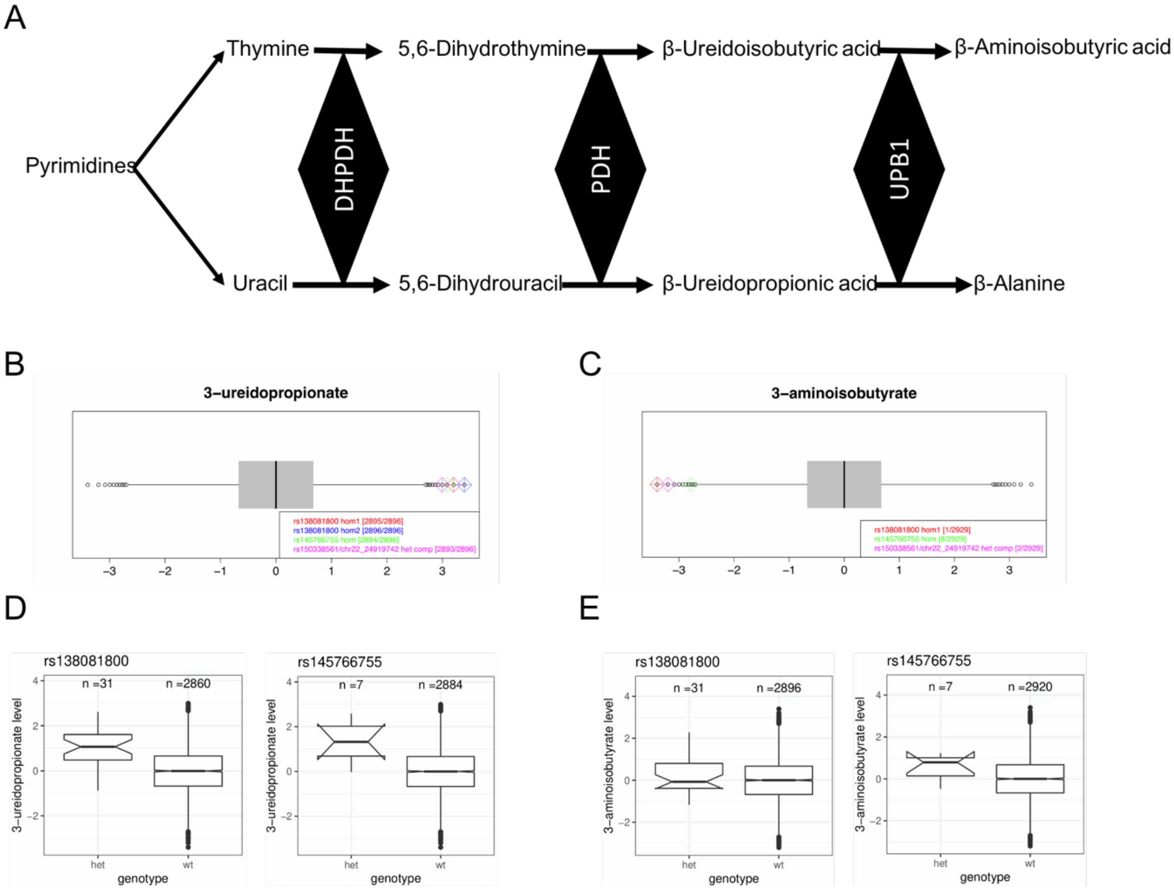

Figure vignette 10. Carriers of UBP1 variants have high levels of beta-ureidopropionic acid and low levels of beta-aminoisobutyratic acid A) Catabolic pathway for pyrimidines. B) Three UPB1 variant homozygotes and one UPB1 compound-heterozygote have high levels of beta-ureidopropionic acid. C) Two UPB1 variant homozygotes and one UPB1 compound-heterozygote have low levels of beta-aminoisobutyric acid. D) Heterozygotes for both UPB1 variants have higher levels of beta-ureidopropionic acid than wild-type individuals. E) No difference was observed between individuals heterozygous for UPB1 variants or not carrying these variants.

Vignette 11: AOX1 and pyridoxate/ methylnicotinamide

We identified three homozygotes for AOX1 variants with low pyridoxate, high methylnicotinamide and low N1-methyl-2-pyridone-5-carboxamide levels (two had missing homozygosity for N1-methyl-2-pyridone-5-carboxamide). AOX1 is involved in the metabolism of many drugs and xenobiotics, and is therefore of interest as a potential drug target. Individual genetic screening for AOX1 variants should be considered when determining the drug dose to be administered.

Aldehyde oxidase 1 (AOX1) is involved in the catabolism of pyridoxal, generating pyridoxic acid (Figure vignette 11A). It is also involved in the degradation of 1-methylnicotinamide to N1-methyl-2-pyridone-5-carboxamide (Figure vignette 11B). We identified one AOX1 donor splice site variant (rs866541106) carried by three homozygotes in the QBB. These three homozygotes had low pyridoxic acid levels (P = 1.14e-08. Figure vignette 11C). Methylnicotinamide levels were high, but not extreme, in AOX1 variant homozygotes (P = 1.15e-04. Figure vignette 11D). Conversely, N1-methyl-2-pyridone-5-carboxamide levels were low in one homozygote and this metabolite was undetectable in the other two (Figure vignette 11E), probably due to the effects of the variant on the generation of this metabolite (Fisher's P = 1.46e-5, Methods). These associations were confirmed in rs866541106 heterozygotes (Figure vignette 11F).

rs866541106 is a very rare variant, with only one European heterozygote reported in GnomAD. The role of AOX1 in human physiology remains unclear. However, interest in AOX1 as a drug-metabolizing enzyme is increasing due to its effects on various drugs and xenobiotics [107,108]. Hartmann et al. used seven AOX1 genetic variants to classify individuals into three principal

groups: fast-metabolizers, poor-metabolizers and individuals with no effect on the catalytic efficiency of AOX1 [109]. Thirteen AOX1 missense variants have been shown to be associated with AOX1 protein production and enzymatic activity [110,111]. An *in-silico* analysis was recently performed to predict putative phenotypic effects and changes in protein stability for most of the reported functional variants of AOX1 [112].

AXO1 metabolizes various drugs containing aldehydes and the most prevalent nitrogen heterocycles [113]. Carriers of the AOX1 variant identified here display considerable impairment of the degradation of both pyridoxal and 1-methylnicotinamide. Thus, individual genetic screening should be considered, to determine the drug dose to be administered.

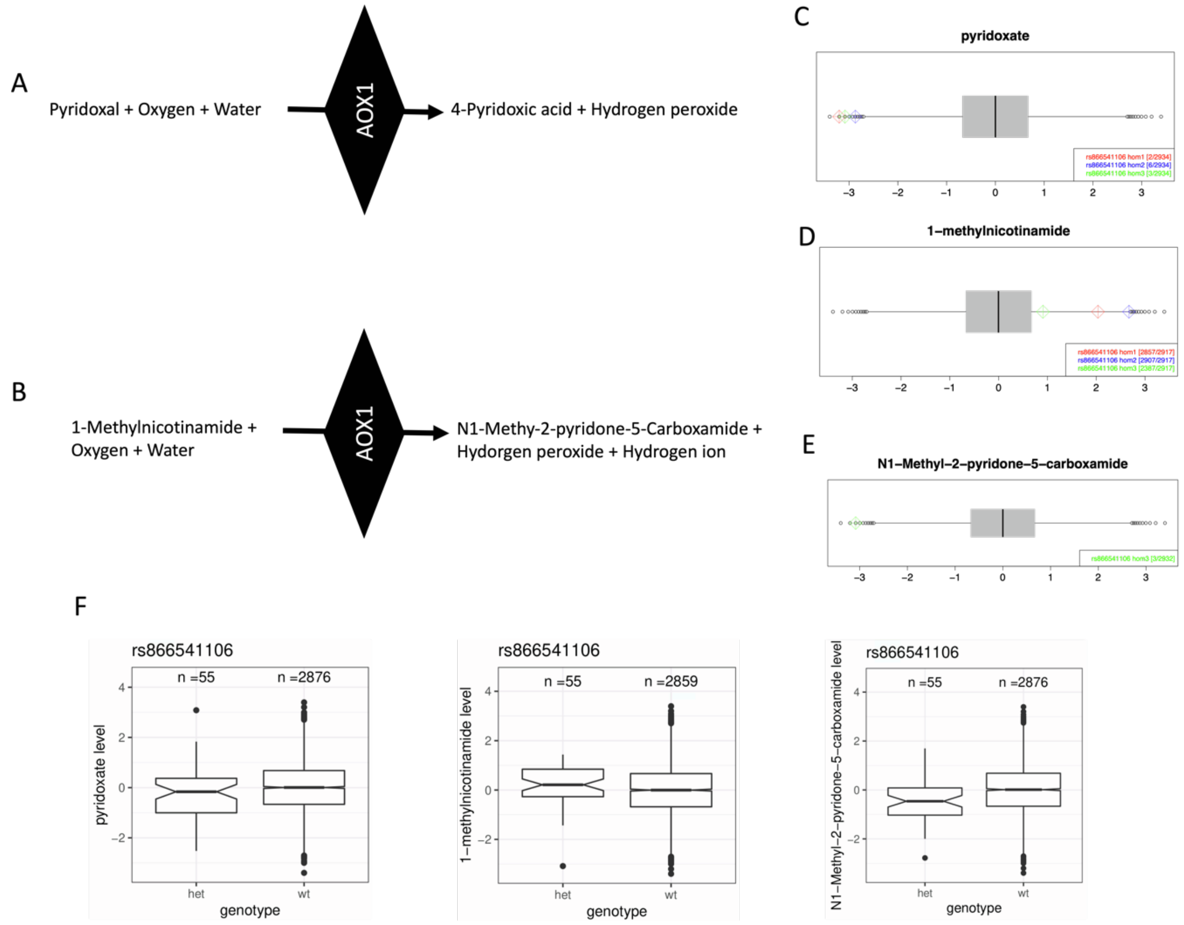

Figure vignette 11. Carriers of the AOX1 donor splice site variant have low levels of pyridoxate and high levels of methylnicotinamide A) Pyridoxal degradation by AOX1. B) Methylnicotinamide degradation by AOX1. C) Three homozygotes for the AOX1 donor splice site variant rs866541106 have low levels of pyridoxic acid. D) Homozygotes for the AOX1 variant have high, but not extreme levels of methylnicotinamide. E) One of the three AOX1 variant carriers has low levels of N1-methyl-2-pyridone-5-carboxamide. N1-methyl-2-pyridone-5-carboxamide was not detected in the other two carriers. F) Heterozygous carriers of rs866541106 have higher levels of methylnicotinamide, lower levels of N1-methyl-2-pyridone-5-carboxamide and similar levels of pyridoxic acid to wild-type individuals.

Vignette 12: ALOX15, PUFAs and asthma

We identified one ALOX15 variant with high levels of linolic and arachidonic acids. This participant also had high levels of other PUFAs, SFAs and various long-chain FAs. This homozygote self-reported taking salbutamol for the treatment of asthma, possibly due to the lack of SPM, the product of PUFA metabolism by ALOX15.

Arachidonate 15-lipoxygenase (ALOX15) is an enzyme responsible for the metabolism of polyunsaturated fatty acids (PUFA) that acts preferentially on linoleic and arachidonic acids [114]. We identified one ALOX15 missense variant, rs41432647, carried by one homozygote in the QBB. Both arachidonic acid and linoleic acid levels were high in the individual homozygous for the ALOX15 variant. The levels of other omega-6 and omega-3 PUFAs were also high in this individual, as were the levels of saturated fatty acids and long-chain fatty acids (Table S8).

ALOX15 is strongly expressed in human airway epithelial cells, mast cells, and eosinophils [115]. Another ALOX15 missense variant, rs34210653, leading to an almost complete loss of enzymatic activity [116,117], decreases the number of circulating eosinophils [118] and confers significant protection against nasal polyps and chronic rhinosinusitis in genome-wide studies [119]. However, this variant is not associated with the risk of asthma [119].

Five homozygotes for the ALOX15 missense variant identified here (rs41432647) were present in the UKB (minor allele frequency = 0.33%). rs41432647 was not significantly associated with eosinophil percentage (P = 0.04), eosinophil count (P = 0.05), risk of nasal polyps (P = 0.8), and doctor-diagnosed asthma (P = 0.28) in the UKB. The rs41432647 homozygote in the QBB had a high percentage and number of eosinophils, ranked 2,681/2,789 and 2,555.5/2,786,

respectively. These observations suggest different effects of the two missense variants, rs41432647 and rs34210653, on ALOX15 enzymatic activity.

The QBB homozygote for rs41432647 was reported to be on salbutamol treatment for asthma and an unspecified allergy. These diseases may stem from the lack of metabolites generated by ALOX15-mediated PUFA metabolism. For instance, the metabolism of PUFA by ALOX15 generates specialized pro-resolving mediators, including lipoxin, resolvin, and protectin. These anti-inflammatory metabolites are involved in the inhibition and fixation of diverse diseases, including asthma [120,121], various inflammatory diseases and other pathogen-induced inflammatory responses, insulin resistance and Alzheimer's disease [122–124]. Further functional analyses are required to assess the potential of ALOX15 inhibition as a treatment target for controlling nasal polyps and chronic rhinosinusitis [119] and the possible role of ALOX15 in asthma development.

Supporting citations

67. Kathiresan, S., Melander, O., Guiducci, C., Surti, A., Burtt, N.P., Rieder, M.J., Cooper, G.M., Roos, C., Voight, B.F., Havulinna, A.S., et al. (2008). Six new loci associated with blood low-density lipoprotein cholesterol, high-density lipoprotein cholesterol or triglycerides in humans. Nat. Genet. *40*, 189–197. 10.1038/ng.75.

68. Qiu, C., Zeng, P., Li, X., Zhang, Z., Pan, B., Peng, Z.Y.F., Li, Y., Ma, Y., Leng, Y., and Chen, R. (2017). What is the impact of PCSK9 rs505151 and rs11591147 polymorphisms on serum lipids level and cardiovascular risk: a meta-analysis. Lipids Health Dis. *16*, 111. 10.1186/s12944-017-0506-6.

69. Verbeek, R., Boyer, M., Boekholdt, S.M., Hovingh, G.K., Kastelein, J.J.P., Wareham, N., Khaw, K.-T., and Arsenault, B.J. (2017). Carriers of the PCSK9 R46L Variant Are Characterized by an Antiatherogenic Lipoprotein Profile Assessed by Nuclear Magnetic Resonance Spectroscopy-Brief Report. Arterioscler. Thromb. Vasc. Biol. *37*, 43–48. 10.1161/ATVBAHA.116.307995.

70. Rao, A.S., Lindholm, D., Rivas, M.A., Knowles, J.W., Montgomery, S.B., and Ingelsson, E. (2018). Large-Scale Phenome-Wide Association Study of PCSK9 Variants Demonstrates Protection Against Ischemic Stroke. Circ. Genomic Precis. Med. *11*, e002162. 10.1161/CIRCGEN.118.002162.

71. Lu, X., Peloso, G.M., Liu, D.J., Wu, Y., Zhang, H., Zhou, W., Li, J., Tang, C.S.-M., Dorajoo, R., Li, H., et al. (2017). Exome chip meta-analysis identifies novel loci and East Asian-specific coding variants that contribute to lipid levels and coronary artery disease. Nat. Genet. *49*, 1722–1730. 10.1038/ng.3978.

72. de Franchis, R., Kraus, E., Kozich, V., Sebastio, G., and Kraus, J.P. (1999). Four novel mutations in the cystathionine beta-synthase gene: effect of a second linked mutation on the severity of the homocystinuric phenotype. Hum. Mutat. *13*, 453–457. 10.1002/(SICI)1098-1004(1999)13:6<453::AID-HUMU4>3.0.CO;2-K.

73. Lee, S.-J., Lee, D.H., Yoo, H.-W., Koo, S.K., Park, E.-S., Park, J.-W., Lim, H.G., and Jung, S.-C. (2005). Identification and functional analysis of cystathionine beta-synthase gene mutations in patients with homocystinuria. J. Hum. Genet. *50*, 648–654. 10.1007/s10038-005-0312-2.

74. El-Said, M.F., Badii, R., Bessisso, M.S., Shahbek, N., El-Ali, M.G., El-Marikhie, M., El-Zyoid, M., Salem, M.S.Z., Bener, A., Hoffmann, G.F., et al. (2006). A common mutation in the CBS gene explains a high incidence of homocystinuria in the Qatari population. Hum. Mutat. *27*, 719. 10.1002/humu.9436.

75. Zschocke, J., Kebbewar, M., Gan-Schreier, H., Fischer, C., Fang-Hoffmann, J., Wilrich, J., Abdoh, G., Ben-Omran, T., Shahbek, N., Lindner, M., et al. (2009). Molecular neonatal screening for homocystinuria in the Qatari population. Hum. Mutat. *30*, 1021–1022. 10.1002/humu.20994.

76. Ageno, W., Gallus, A.S., Wittkowsky, A., Crowther, M., Hylek, E.M., and Palareti, G. (2012). Oral anticoagulant therapy: Antithrombotic Therapy and Prevention of Thrombosis, 9th ed: American College of Chest Physicians Evidence-Based Clinical Practice Guidelines. Chest *141*, e44S-e88S. 10.1378/chest.11-2292.

77. Gately, S., Twardowski, P., Stack, M.S., Cundiff, D.L., Grella, D., Castellino, F.J., Enghild, J., Kwaan, H.C., Lee, F., Kramer, R.A., et al. (1997). The mechanism of cancer-mediated conversion of plasminogen to the angiogenesis inhibitor angiostatin. Proc. Natl. Acad. Sci. U. S. A. *94*, 10868–10872. 10.1073/pnas.94.20.10868.

78. Dodd, D., Spitzer, M.H., Van Treuren, W., Merrill, B.D., Hryckowian, A.J., Higginbottom, S.K., Le, A., Cowan, T.M., Nolan, G.P., Fischbach, M.A., et al. (2017). A gut bacterial pathway metabolizes aromatic amino acids into nine circulating metabolites. Nature *551*, 648–652. 10.1038/nature24661.

79. Elsden, S.R., Hilton, M.G., and Waller, J.M. (1976). The end products of the metabolism of aromatic amino acids by Clostridia. Arch. Microbiol. *107*, 283–288. 10.1007/BF00425340.

80. Masters, C.L., Simms, G., Weinman, N.A., Multhaup, G., McDonald, B.L., and Beyreuther, K. (1985). Amyloid plaque core protein in Alzheimer disease and Down syndrome. Proc. Natl. Acad. Sci. U. S. A. *82*, 4245–4249. 10.1073/pnas.82.12.4245.

81. Bendheim, P.E., Poeggeler, B., Neria, E., Ziv, V., Pappolla, M.A., and Chain, D.G. (2002). Development of indole-3-propionic acid (OXIGON) for Alzheimer's disease. J. Mol. Neurosci. MN *19*, 213–217. 10.1007/s12031-002-0036-0.

82. Chyan, Y.J., Poeggeler, B., Omar, R.A., Chain, D.G., Frangione, B., Ghiso, J., and Pappolla, M.A. (1999). Potent neuroprotective properties against the Alzheimer beta-amyloid by an endogenous melatonin-related indole structure, indole-3-propionic acid. J. Biol. Chem. *274*, 21937–21942. 10.1074/jbc.274.31.21937.

83. Karbownik, M., Stasiak, M., Zygmunt, A., Zasada, K., and Lewiński, A. (2006). Protective effects of melatonin and indole-3-propionic acid against lipid peroxidation, caused by potassium bromate in the rat kidney. Cell Biochem. Funct. *24*, 483–489. 10.1002/cbf.1321.

84. Venkatesh, M., Mukherjee, S., Wang, H., Li, H., Sun, K., Benechet, A.P., Qiu, Z., Maher, L., Redinbo, M.R., Phillips, R.S., et al. (2014). Symbiotic bacterial metabolites regulate gastrointestinal barrier function via the xenobiotic sensor PXR and Toll-like receptor 4. Immunity *41*, 296–310. 10.1016/j.immuni.2014.06.014.

85. Zhao, Z.-H., Xin, F.-Z., Xue, Y., Hu, Z., Han, Y., Ma, F., Zhou, D., Liu, X.-L., Cui, A., Liu, Z., et al. (2019). Indole-3-propionic acid inhibits gut dysbiosis and endotoxin leakage to attenuate steatohepatitis in rats. Exp. Mol. Med. *51*, 1–14. 10.1038/s12276-019-0304-5.

86. de Mello, V.D., Paananen, J., Lindström, J., Lankinen, M.A., Shi, L., Kuusisto, J., Pihlajamäki, J., Auriola, S., Lehtonen, M., Rolandsson, O., et al. (2017). Indolepropionic

acid and novel lipid metabolites are associated with a lower risk of type 2 diabetes in the Finnish Diabetes Prevention Study. Sci. Rep. *7*, 46337. 10.1038/srep46337.

87. Tuomainen, M., Lindström, J., Lehtonen, M., Auriola, S., Pihlajamäki, J., Peltonen, M., Tuomilehto, J., Uusitupa, M., de Mello, V.D., and Hanhineva, K. (2018). Associations of serum indolepropionic acid, a gut microbiota metabolite, with type 2 diabetes and low-grade inflammation in high-risk individuals. Nutr. Diabetes *8*, 35. 10.1038/s41387-018-0046-9.

88. Connor, W.E., Lin, D.S., Pappu, A.S., Frohlich, J., and Gerhard, G. (2005). Dietary sitostanol and campestanol: accumulation in the blood of humans with sitosterolemia and xanthomatosis and in rat tissues. Lipids *40*, 919–923. 10.1007/s11745-005-1452-7.

89. Williams, K., Segard, A., and Graf, G.A. (2021). Sitosterolemia: Twenty Years of Discovery of the Function of ABCG5ABCG8. Int. J. Mol. Sci. *22*, 2641. 10.3390/ijms22052641.

90. Wang, H.H., Liu, M., Portincasa, P., and Wang, D.Q.-H. (2020). Recent Advances in the Critical Role of the Sterol Efflux Transporters ABCG5/G8 in Health and Disease. Adv. Exp. Med. Biol. *1276*, 105–136. 10.1007/978-981-15-6082-8_8.

91. Portincasa, P., Di Ciaula, A., de Bari, O., Garruti, G., Palmieri, V.O., and Wang, D.Q.-H. (2016). Management of gallstones and its related complications. Expert Rev. Gastroenterol. Hepatol. *10*, 93–112. 10.1586/17474124.2016.1109445.

92. Kajinami, K., Brousseau, M.E., Nartsupha, C., Ordovas, J.M., and Schaefer, E.J. (2004). ATP binding cassette transporter G5 and G8 genotypes and plasma lipoprotein levels before and after treatment with atorvastatin. J. Lipid Res. *45*, 653–656. 10.1194/jlr.M300278-JLR200.

93. Gylling, H., Hallikainen, M., Pihlajamäki, J., Agren, J., Laakso, M., Rajaratnam, R.A., Rauramaa, R., and Miettinen, T.A. (2004). Polymorphisms in the ABCG5 and ABCG8 genes associate with cholesterol absorption and insulin sensitivity. J. Lipid Res. *45*, 1660–1665. 10.1194/jlr.M300522-JLR200.

94. Berge, K.E., von Bergmann, K., Lutjohann, D., Guerra, R., Grundy, S.M., Hobbs, H.H., and Cohen, J.C. (2002). Heritability of plasma noncholesterol sterols and relationship to DNA sequence polymorphism in ABCG5 and ABCG8. J. Lipid Res. *43*, 486–494.

95. Kuo, K.-K., Shin, S.-J., Chen, Z.-C., Yang, Y.-H.C., Yang, J.-F., and Hsiao, P.-J. (2008). Significant association of ABCG5 604Q and ABCG8 D19H polymorphisms with gallstone disease. Br. J. Surg. *95*, 1005–1011. 10.1002/bjs.6178.

96. Katsika, D., Magnusson, P., Krawczyk, M., Grünhage, F., Lichtenstein, P., Einarsson, C., Lammert, F., and Marschall, H.-U. (2010). Gallstone disease in Swedish twins: risk is associated with ABCG8 D19H genotype. J. Intern. Med. *268*, 279–285. 10.1111/j.1365-2796.2010.02249.x.

97. Yu, L., Hammer, R.E., Li-Hawkins, J., Von Bergmann, K., Lutjohann, D., Cohen, J.C., and Hobbs, H.H. (2002). Disruption of Abcg5 and Abcg8 in mice reveals their crucial role in

biliary cholesterol secretion. Proc. Natl. Acad. Sci. U. S. A. *99*, 16237–16242. 10.1073/pnas.252582399.

98. Alonso, A., Yu, B., Qureshi, W.T., Grams, M.E., Selvin, E., Soliman, E.Z., Loehr, L.R., Chen, L.Y., Agarwal, S.K., Alexander, D., et al. (2015). Metabolomics and Incidence of Atrial Fibrillation in African Americans: The Atherosclerosis Risk in Communities (ARIC) Study. PloS One *10*, e0142610. 10.1371/journal.pone.0142610.

99. Gimenez, F., Fernandez, C., and Mabondzo, A. (2004). Transport of HIV protease inhibitors through the blood-brain barrier and interactions with the efflux proteins, P-glycoprotein and multidrug resistance proteins. J. Acquir. Immune Defic. Syndr. 1999 *36*, 649–658. 10.1097/00126334-200406010-00001.

100. Weiss, J., Theile, D., Ketabi-Kiyanvash, N., Lindenmaier, H., and Haefeli, W.E. (2007). Inhibition of MRP1/ABCC1, MRP2/ABCC2, and MRP3/ABCC3 by nucleoside, nucleotide, and non-nucleoside reverse transcriptase inhibitors. Drug Metab. Dispos. Biol. Fate Chem. *35*, 340–344. 10.1124/dmd.106.012765.

101. Vujcic, S., Liang, P., Diegelman, P., Kramer, D.L., and Porter, C.W. (2003). Genomic identification and biochemical characterization of the mammalian polyamine oxidase involved in polyamine back-conversion. Biochem. J. *370*, 19–28. 10.1042/BJ20021779.

102. Hugill, A.J., Stewart, M.E., Yon, M.A., Probert, F., Cox, I.J., Hough, T.A., Scudamore, C.L., Bentley, L., Wall, G., Wells, S.E., et al. (2015). Loss of arylformamidase with reduced thymidine kinase expression leads to impaired glucose tolerance. Biol. Open *4*, 1367–1375. 10.1242/bio.013342.

103. Moolenaar, S.H., Göhlich-Ratmann, G., Engelke, U.F., Spraul, M., Humpfer, E., Dvortsak, P., Voit, T., Hoffmann, G.F., Bräutigam, C., van Kuilenburg, A.B., et al. (2001). beta-Ureidopropionase deficiency: a novel inborn error of metabolism discovered using NMR spectroscopy on urine. Magn. Reson. Med. *46*, 1014–1017. 10.1002/mrm.1289.

104. Kölker, S., Okun, J.G., Hörster, F., Assmann, B., Ahlemeyer, B., Kohlmüller, D., Exner-Camps, S., Mayatepek, E., Krieglstein, J., and Hoffmann, G.F. (2001). 3-Ureidopropionate contributes to the neuropathology of 3-ureidopropionase deficiency and severe propionic aciduria: a hypothesis. J. Neurosci. Res. *66*, 666–673. 10.1002/jnr.10012.

105. van Kuilenburg, A.B.P., Meinsma, R., Beke, E., Assmann, B., Ribes, A., Lorente, I., Busch, R., Mayatepek, E., Abeling, N.G.G.M., van Cruchten, A., et al. (2004). beta-Ureidopropionase deficiency: an inborn error of pyrimidine degradation associated with neurological abnormalities. Hum. Mol. Genet. *13*, 2793–2801. 10.1093/hmg/ddh303.

106. van Kuilenburg, A.B.P., Dobritzsch, D., Meijer, J., Krumpel, M., Selim, L.A., Rashed, M.S., Assmann, B., Meinsma, R., Lohkamp, B., Ito, T., et al. (2012). ß-ureidopropionase deficiency: phenotype, genotype and protein structural consequences in 16 patients. Biochim. Biophys. Acta *1822*, 1096–1108. 10.1016/j.bbadis.2012.04.001.

107. Pryde, D.C., Dalvie, D., Hu, Q., Jones, P., Obach, R.S., and Tran, T.-D. (2010). Aldehyde oxidase: an enzyme of emerging importance in drug discovery. J. Med. Chem. *53*, 8441–8460. 10.1021/jm100888d.

108. Smith, M.A., Marinaki, A.M., Arenas, M., Shobowale-Bakre, M., Lewis, C.M., Ansari, A., Duley, J., and Sanderson, J.D. (2009). Novel pharmacogenetic markers for treatment outcome in azathioprine-treated inflammatory bowel disease. Aliment. Pharmacol. Ther. *30*, 375–384. 10.1111/j.1365-2036.2009.04057.x.

109. Hartmann, T., Terao, M., Garattini, E., Teutloff, C., Alfaro, J.F., Jones, J.P., and Leimkühler, S. (2012). The impact of single nucleotide polymorphisms on human aldehyde oxidase. Drug Metab. Dispos. Biol. Fate Chem. *40*, 856–864. 10.1124/dmd.111.043828.

110. Foti, A., Hartmann, T., Coelho, C., Santos-Silva, T., Romão, M.J., and Leimkühler, S. (2016). Optimization of the Expression of Human Aldehyde Oxidase for Investigations of Single-Nucleotide Polymorphisms. Drug Metab. Dispos. Biol. Fate Chem. *44*, 1277–1285. 10.1124/dmd.115.068395.

111. Foti, A., Dorendorf, F., and Leimkühler, S. (2017). A single nucleotide polymorphism causes enhanced radical oxygen species production by human aldehyde oxidase. PloS One *12*, e0182061. 10.1371/journal.pone.0182061.

112. Coelho, C., Muthukumaran, J., Santos-Silva, T., and João Romão, M. (2019). Systematic exploration of predicted destabilizing nonsynonymous single nucleotide polymorphisms (nsSNPs) of human aldehyde oxidase: A Bio-informatics study. Pharmacol. Res. Perspect. *7*, e00538. 10.1002/prp2.538.

113. Torres, R.A., Korzekwa, K.R., McMasters, D.R., Fandozzi, C.M., and Jones, J.P. (2007). Use of density functional calculations to predict the regioselectivity of drugs and molecules metabolized by aldehyde oxidase. J. Med. Chem. *50*, 4642–4647. 10.1021/jm0703690.

114. Feltenmark, S., Gautam, N., Brunnström, A., Griffiths, W., Backman, L., Edenius, C., Lindbom, L., Björkholm, M., and Claesson, H.-E. (2008). Eoxins are proinflammatory arachidonic acid metabolites produced via the 15-lipoxygenase-1 pathway in human eosinophils and mast cells. Proc. Natl. Acad. Sci. U. S. A. *105*, 680–685. 10.1073/pnas.0710127105.

115. Claesson, H.-E. (2009). On the biosynthesis and biological role of eoxins and 15-lipoxygenase-1 in airway inflammation and Hodgkin lymphoma. Prostaglandins Other Lipid Mediat. *89*, 120–125. 10.1016/j.prostaglandins.2008.12.003.

116. Assimes, T.L., Knowles, J.W., Priest, J.R., Basu, A., Borchert, A., Volcik, K.A., Grove, M.L., Tabor, H.K., Southwick, A., Tabibiazar, R., et al. (2008). A near null variant of 12/15-LOX encoded by a novel SNP in ALOX15 and the risk of coronary artery disease. Atherosclerosis *198*, 136–144. 10.1016/j.atherosclerosis.2007.09.003.

117. Schurmann, K., Anton, M., Ivanov, I., Richter, C., Kuhn, H., and Walther, M. (2011). Molecular basis for the reduced catalytic activity of the naturally occurring T560M mutant of human 12/15-lipoxygenase that has been implicated in coronary artery disease. J. Biol. Chem. *286*, 23920–23927. 10.1074/jbc.M110.211821.

118. Astle, W.J., Elding, H., Jiang, T., Allen, D., Ruklisa, D., Mann, A.L., Mead, D., Bouman, H., Riveros-Mckay, F., Kostadima, M.A., et al. (2016). The Allelic Landscape of Human Blood Cell Trait Variation and Links to Common Complex Disease. Cell *167*, 1415-1429.e19. 10.1016/j.cell.2016.10.042.

119. Kristjansson, R.P., Benonisdottir, S., Davidsson, O.B., Oddsson, A., Tragante, V., Sigurdsson, J.K., Stefansdottir, L., Jonsson, S., Jensson, B.O., Arthur, J.G., et al. (2019). A loss-of-function variant in ALOX15 protects against nasal polyps and chronic rhinosinusitis. Nat. Genet. *51*, 267–276. 10.1038/s41588-018-0314-6.

120. Barnig, C., and Levy, B.D. (2015). Innate immunity is a key factor for the resolution of inflammation in asthma. Eur. Respir. Rev. Off. J. Eur. Respir. Soc. *24*, 141–153. 10.1183/09059180.00012514.

121. Rogerio, A.P., Haworth, O., Croze, R., Oh, S.F., Uddin, M., Carlo, T., Pfeffer, M.A., Priluck, R., Serhan, C.N., and Levy, B.D. (2012). Resolvin D1 and aspirin-triggered resolvin D1 promote resolution of allergic airways responses. J. Immunol. Baltim. Md 1950 *189*, 1983–1991. 10.4049/jimmunol.1101665.

122. Cole, B.K., Lieb, D.C., Dobrian, A.D., and Nadler, J.L. (2013). 12- and 15-lipoxygenases in adipose tissue inflammation. Prostaglandins Other Lipid Mediat. *104–105*, 84–92. 10.1016/j.prostaglandins.2012.07.004.

123. Qu, Q., Xuan, W., and Fan, G.-H. (2015). Roles of resolvins in the resolution of acute inflammation. Cell Biol. Int. *39*, 3–22. 10.1002/cbin.10345.

124. Heras-Sandoval, D., Pedraza-Chaverri, J., and Pérez-Rojas, J.M. (2016). Role of docosahexaenoic acid in the modulation of glial cells in Alzheimer’s disease. J. Neuroinflammation *13*, 61. 10.1186/s12974-016-0525-7.
